# A Middle Pleistocene wolf from central Italy provides insights on the first occurrence of *Canis lupus* in Europe

**DOI:** 10.1038/s41598-022-06812-5

**Published:** 2022-02-25

**Authors:** Dawid A. Iurino, Beniamino Mecozzi, Alessio Iannucci, Alfio Moscarella, Flavia Strani, Fabio Bona, Mario Gaeta, Raffaele Sardella

**Affiliations:** 1 PaleoFactory, Sapienza Università di Roma, Piazzale Aldo Moro 5, 00185 Rome, Italy; 2 Dipartimento di Scienze della Terra, Sapienza Università di Roma, Piazzale Aldo Moro 5, 00185 Rome, Italy; 3 Dipartimento di Scienze della Terra “A. Desio”, Via Mangiagalli 34, 20133 Milan, Italy

**Keywords:** Palaeontology, Evolution, Zoology

## Abstract

Here, we describe a partial cranium of a large canid dated at 406.5 ± 2.4 ka from the Middle Pleistocene of Ponte Galeria (Rome, Italy). The sample represents one of the few Middle Pleistocene remains of a wolf-like canid falling within the timeframe when the *Canis mosbachensis*–*Canis lupus* transition occurred, a key moment to understand the spread of the extant wolf (*Canis lupus*) in Europe. CT-based methods allow studying the outer and inner cranial anatomy (brain and frontal sinuses) of a selected sample of fossil and extant canids. Morphological and biometric results allowed to: (I) ascribe the cranium from Ponte Galeria to an adult *Canis lupus*, representing the first reliable occurrence of this taxon in Europe; (II) provide the content for a biochronological revision of the Middle Pleistocene record of European wolves.

## Introduction

The ecological plasticity, the pack hunting abilities and the complex social structure are just some of the traits that contribute to making the wolf (*Canis lupus*, Linnaeus, 1758) the most widespread and iconic representative of the genus *Canis*, with a considerable impact on the collective human imagination over the centuries^1^. The European fossil record of Late Pleistocene *C. lupus* is remarkable in terms of number of remains and their preservation, offering a privileged context for investigating the paleobiology of the wolf^2–5^, as well as the early human-wolf interactions and the origin of the domestic dog^6–9^. Moving back to the Middle Pleistocene, our knowledge on the origin of *C. lupus* in Europe is instead limited due to the paucity of fossil remains^10,11^.

**Figure 1:**
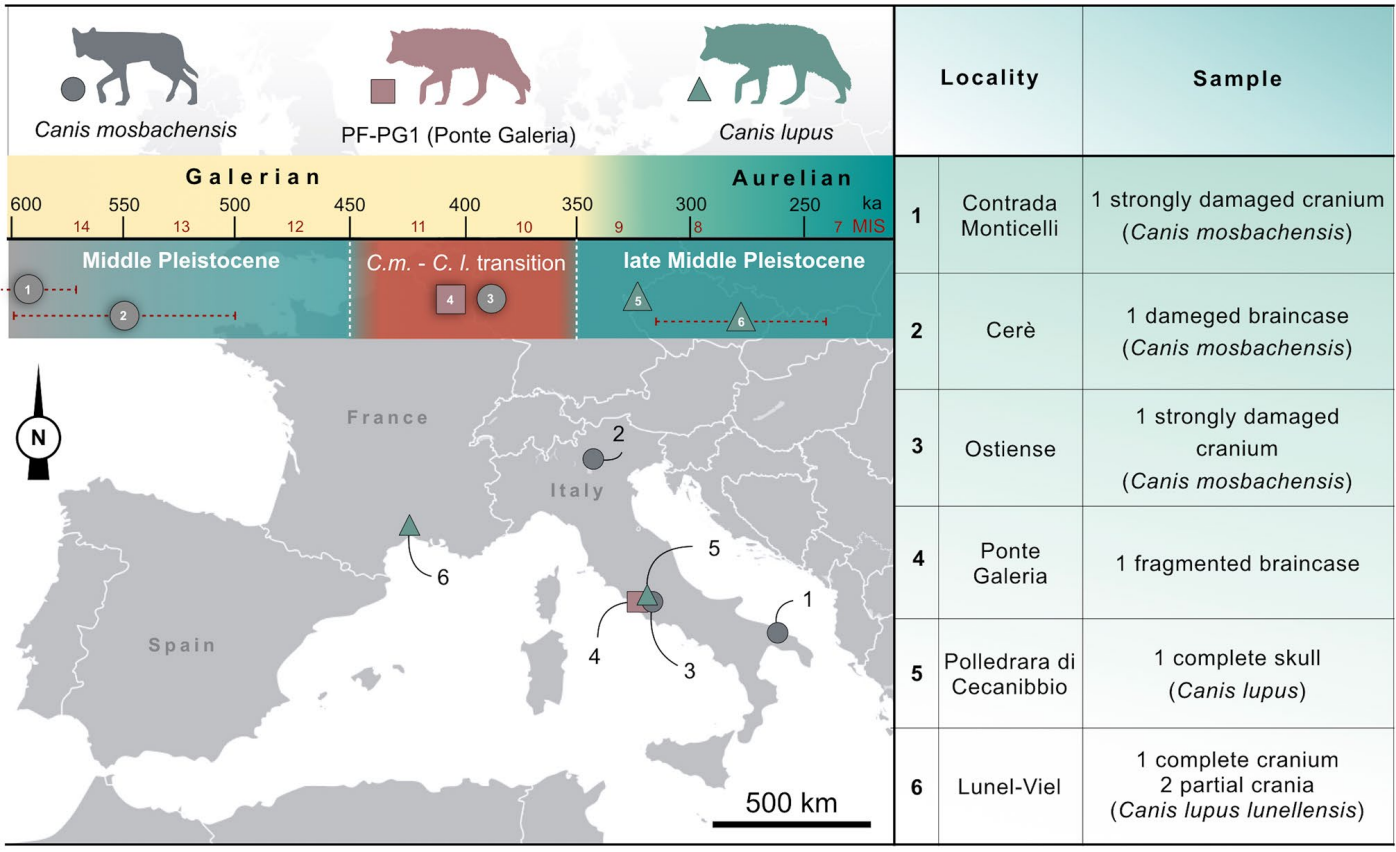
Cranial remains of Middle Pleistocene wolf-like canids from Europe at the time of the *Canis mosbachensis*–*Canis lupus* transition (for the complete list see Supplementary Table S1). Map of Europe modified from https://en.wikipedia.org/wiki/File:Europe_blank_map.png, artwork by D.A. Iurino.

Although there are a few remains attributed to *C. lupus* from the late Early Pleistocene of North America^12^, there is broad consensus in considering the Early to Middle Pleistocene Mosbach wolf (*Canis mosbachensis*, Soergel, 1925) the ancestor of *C. lupus*, with a replacement occurred in Europe during the Middle Pleistocene ca. 450–350 ka (MIS 12-MIS 9)^11–13^. The first occurrence (FO) of the modern wolf is one of the defining bioevents of the faunal turnover that took place during the transition between the Galerian and Aurelian European Land Mammal Ages (ca. 400 ka), characterized by the earliest dispersal of several modern mammal taxa in Europe^14–17^. Despite being intensively investigated from taxonomic, morphological and biochronological perspectives, European Middle Pleistocene wolves have been attributed either to *C. mosbachensis* or *C. lupus* mainly due to their size^4,13,18–21^. An increase in dimensions is usually recorded across the Middle Pleistocene, with the largest *Canis* reported during the end of the Late Pleistocene (*C. l. maximus*, sensu Brugal and Boudadì-Maligne^2^). The morphological differences between *C. mosbachensis* and *C. lupus* have long been vaguely defined (Supplementary Note 1), as the remains of wolf-like canids from the Middle Pleistocene of Europe are mainly of poor taxonomic value (Fig. 1). As pointed out by Mecozzi et al.^10^, some fossil and extant *C. lupus* frequently share *C. mosbachensis*-like characters. Most importantly, there is overlap in tooth size between these two species, leading to uncertain taxonomic attributions. Aside from the Ostiense cranium (456–416 ka, MIS 11)^2^, which represents the last occurrence (LO) of *C. mosbachensis* in Europe, other informative cranial remains far predate the crucial period from MIS 12 to MIS 9, when the *Canis mosbachensis-Canis lupus* transition occurred^11,13,19^.

The earliest customarily accepted record of *Canis lupus* was that of Lunel-Viel (France), referred to MIS 11 (ca. 400 ka) and interpreted as a chronosubspecies of the wolf, *C. lupus lunellensis*^22^. Given the relatively small size of the remains, they would have fitted in a scenario of a progressive size increase in the *C. mosbachensis*–*C. lupus* lineage, being interpreted as one of the last-surviving populations of small-sized wolves^22^—although notably the possibility of a synonymy with the Mosbach wolf has been suggested by Kurtén^23^. However, the estimated antiquity of Lunel-Viel was based on biochronological inference and new studies are pointing to a younger age, likely MIS 9-7^24,25^. During MIS 11-9, canid samples from well temporally constrained deposits of Europe are lacking, with the only exception of the nearly complete skull of *C. lupus* from La Polledrara di Cecanibbio (Rome, Italy) (340–320 ka, MIS 9)^26^. This specimen has not been described yet, although it has been figured and ascribed to *C. lupus* by Anzidei et al.^26^.

In this scenario, the sample (PF-PG1) collected from the area of Ponte Galeria (Rome, Italy) is relevant because it represents one of the few Middle Pleistocene crania of a wolf-like canid and offers an unique opportunity to enrich our knowledge on the *C. mosbachensis*-*C. lupus* transition in Europe. The first CT-based description of the inner and outer cranial anatomy of the specimen from Ponte Galeria is offered here, providing the content for a biochronological revision of the Middle Pleistocene record of European wolves.

### Geological framework

**Figure 2:**
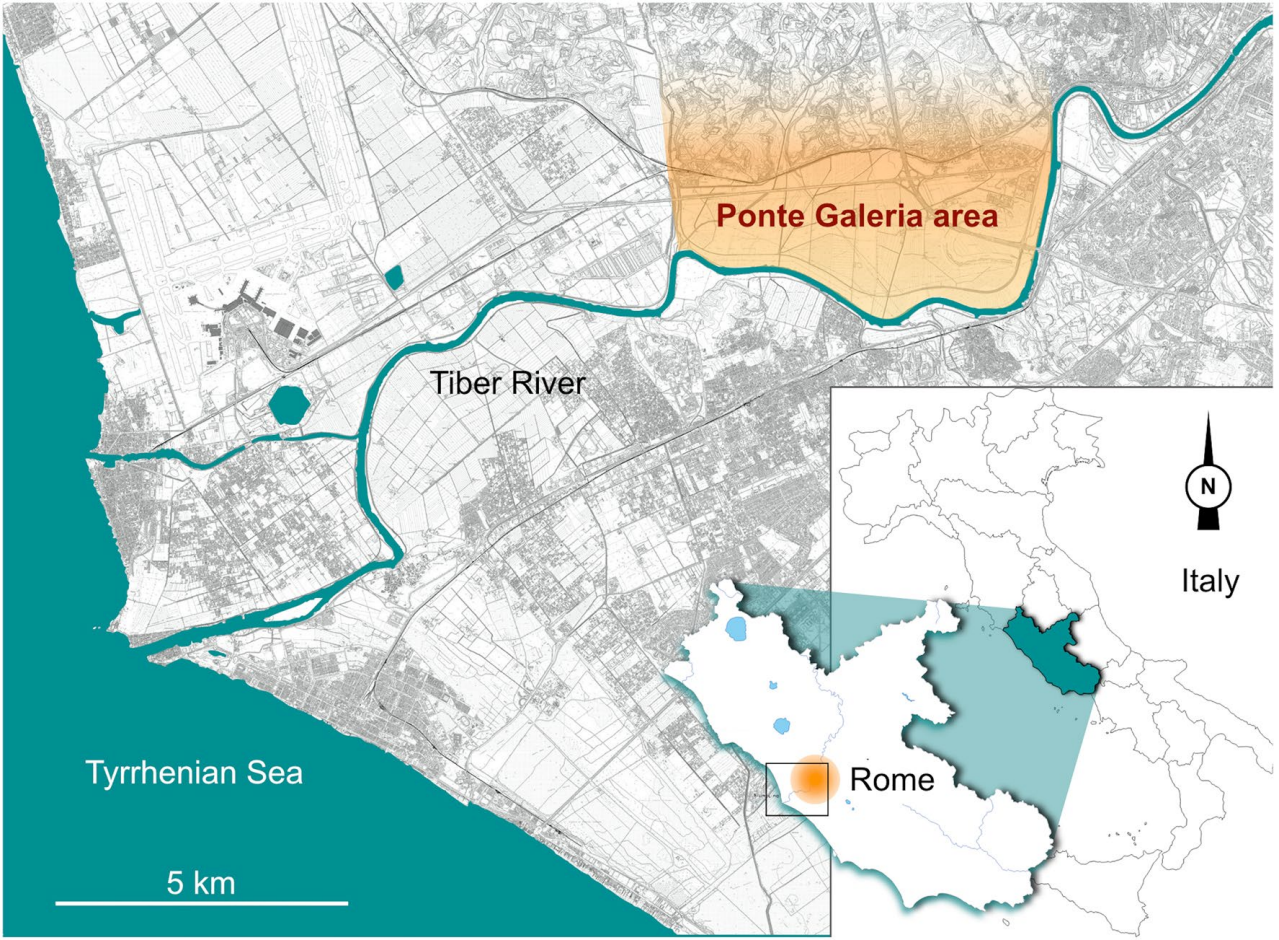
Geographic location of Ponte Galeria area. Map of Italy modified from https://it.wikipedia.org/wiki/File:Italy_map_with_regions.svg, map of Rome modified from the Carta Tecnica Regionale 2014 del Lazio, foglio 386—Lido di Ostia—https://geoportale.regione.lazio.it/layers/geodbgt:geonode:curve_livello#more.

Since the beginning of 1960s, the sedimentary sequence of Ponte Galeria, located in the west area of Rome (Fig. 2), represents one of the richest paleontological localities of the Italian Peninsula. Ponte Galeria is considered a reference for biochronological and paleoecological studies of European Middle Pleistocene faunas, and give its name to the homonym biochronological unit (Ponte Galeria Faunal Unit). The term Galerian, now widely adopted as a Mammal Age, was introduced by Ambrosetti et al.^27^ to define a peculiar late Early–Middle Pleistocene faunal assemblage recovered from the area^14,28^. Here, several fossiliferous localities are known spanning in age from the late Early Pleistocene to the late Middle Pleistocene^29^. Among them, those referred to MIS 11-9 are: Castel di Guido, Fontignano 2, Malagrotta, La Polledrara di Cecanibbio, San Cosimato, Torre in Pietra—lower levels^30–32^. During this time span, proboscideans, hippopotamuses, cervids and bovids are well documented, whereas the record of carnivorans is quite poor. Lower Paleolithic tools were found at Castel di Guido^31,33^, La Polledrara di Cecanibbio^26^, Malagrotta^31^ and Torre in Pietra (lower levels)^34^.

## Results

### Age of the Ponte Galeria wolf

**Figure 3:**
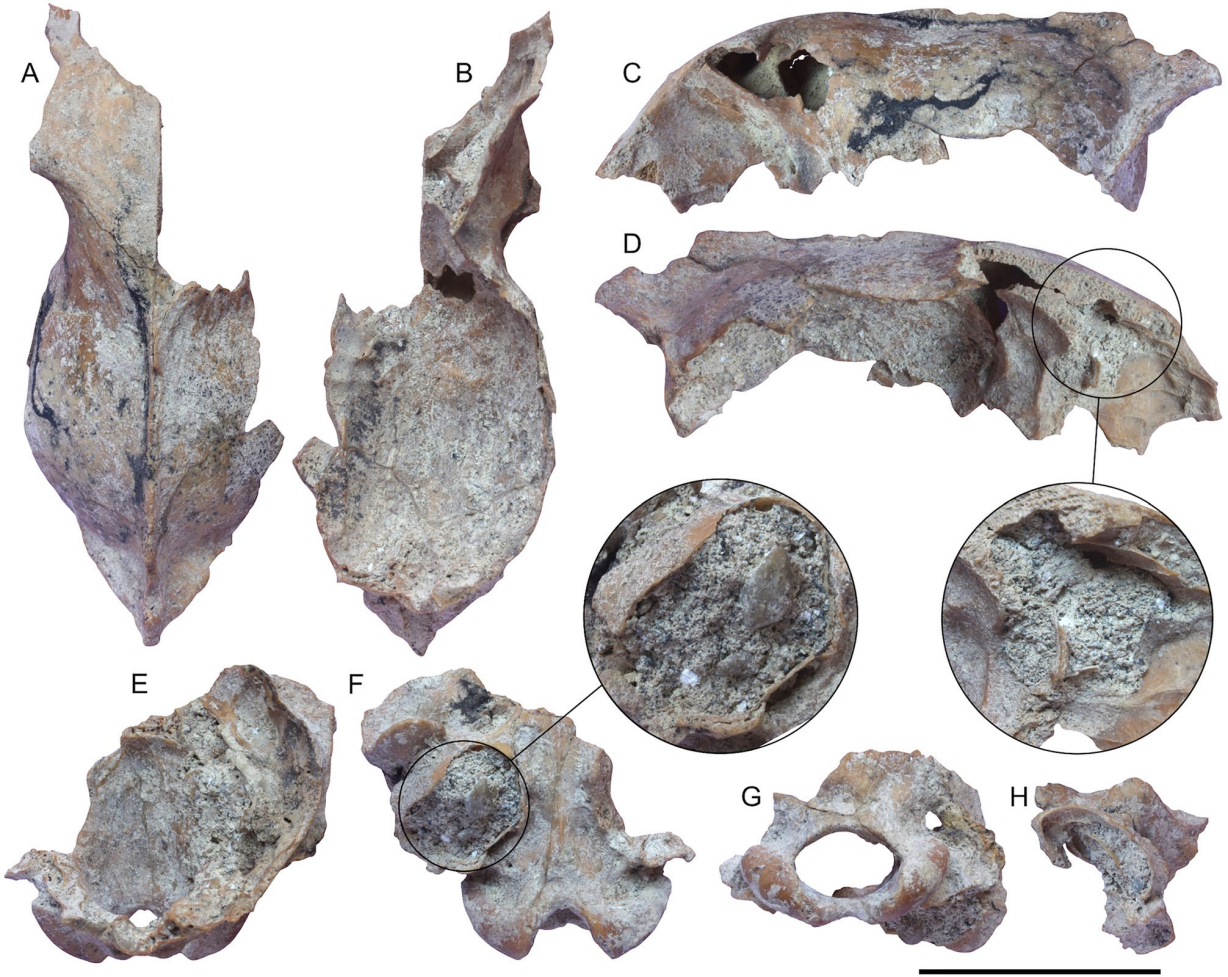
PF-PG1 specimen from Ponte Galeria. Upper portion of the cranium in dorsal (**A**), ventral (**B**), left lateral (**C**) and right lateral (**D**) views, with close-up of the encrusting sediment. Basicranium in dorsal (**E**) and ventral (**F**) views with a close-up of the left tympanic bulla filled by pumice clasts, occipital condyles in posterior view (**G**), right tympanic bulla in ventral view (**H**). Scale bar: 50 mm.

PF-PG1 was recovered during rescue surveys between the 1970s and 1980s. Unfortunately, as for other remains collected during quarry activities in those years, the exact location and stratigraphic provenance of PF-PG1 were not recorded and the specimen is simply labelled as from "Ponte Galeria". However, this region is rich in geochronologically calibrated continental deposits younger than ca. 800 ka^35,36^. The cavities of the cranium are infilled by whitish and highly vesicular pumice clasts (Fig. 3), whose geochemical characterization allows correlation with the Vico β pyroclastites erupted at 406.5 ± 2.4 ka (Supplementary Note 2, Supplementary Figs. S1, S2 and Supplementary Table S2).

### Outer cranial anatomy

**Figure 4:**
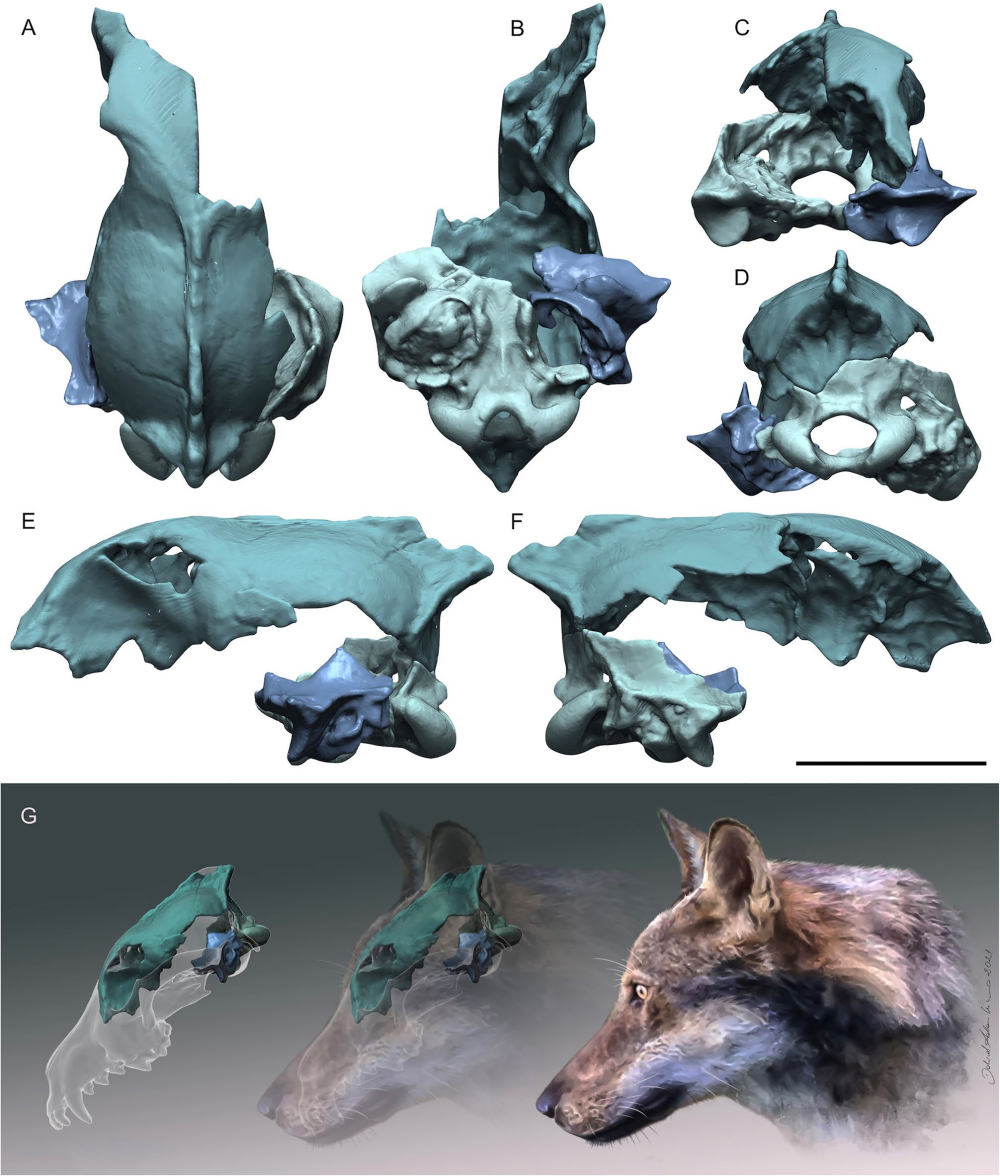
Virtually restored model of PF-PG1 specimen from Ponte Galeria in dorsal (**A**), ventral (**B**), anterior (**C**), posterior (**D**), left lateral and right lateral views. Scale bars: 50 mm. Reconstruction sequence of the head appearance of PF-PG1. Artwork by D.A. Iurino.

The specimen PF-PG1 (Figs. 3, 4) consists of a partial braincase divided into four fragments of different size. The surface of the fragments is covered by a thin and irregular encrusting patina of volcanic ash, light grey in color. The coarse volcanic sediment is mostly located within the tympanic bullae, while it is less abundant inside the frontal bone. The specimen has completely fused cranial sutures, which is compatible with the adult condition, and it shows no signs of bone alterations or transport (weathering or abrasion).

The postorbital constriction is wide (even if preserved only on the left side), the slightly marked left temporal line ending posteriorly to a long and well-developed sagittal crest. The neurocranium is fairly inflated in dorsal view. The frontals are convex and elevated, whereas the sagittal crest is quite robust and the projection posterior end exceeds the occipital condyles in lateral view. The retroarticular process is robust as well the mastoid one, whereas the opening of the acoustic meatus is oval with antero-ventral to postero-dorsal major axis. Posteriorly, the braincase is rounded and has a rough surface, with a marked nuchal crest. The foramen magnum is oval and elongated laterally, the occipital condyles are laterally and dorsally projected and the nuchal tubercle is weak. The tympanic bullae are partially broken. They are antero-medially to postero-laterally elongated, their anterior borders are aligned with the retroarticular processes, and their medial walls are parallel in ventral view. PF-PG1 shows frontal bones slightly convex in lateral view, similarly to those of other fossil and extant specimens of *C. lupus*, but differing from the condition of *C. mosbachensis*, where the frontals are less prominent^10,13^ (Supplementary Fig. S3)*.* The medial wall of the tympanic bullae is divergent in the Ponte Galeria specimen, similarly to those of *C. lupus* and *C. mosbachensis*^13^*.*

### Biometric comparison

**Figure 5:**
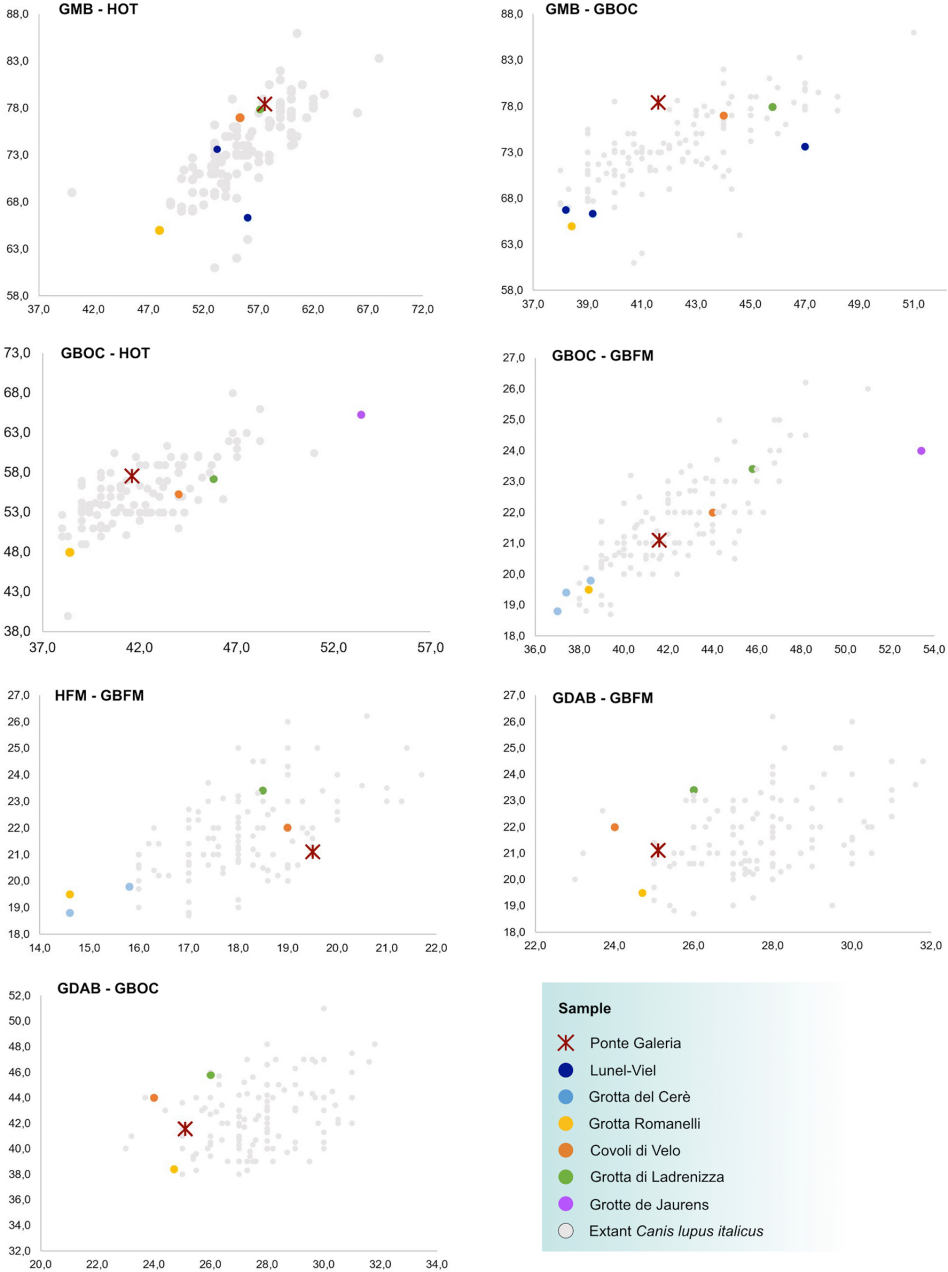
Bivariate plots of cranial measurements of fossil and extant canids. *GDAB* Greatest diameter of the auditory bulla, *GMB* Greatest mastoid breadth, *GBOC* Greatest breadth of the occipital condyles, *GBFM* Greatest breadth of the foramen magnum, *HFM* Height of the foramen magnum, *HOT* Height of the occipital triangle. Measurements are reported in Supplementary Table S3.

Cranial measurements of PF-PG1 (Fig. 5, Supplementary Table S3) falls within the range of extant *C. lupus italicus* for all the considered variables. In the GMB-HOT plot, PF-PG1 is larger than specimens from Lunel-Viel and Grotta Romanelli, ranking close to those of Covoli di Velo, Grotta Ladrenizza and extant *C. lupus italicus* (Fig. 5)*.* A similar arrangement is observable in the GMB-GBOC plot, except for a single specimen from Lunel-Viel which is nearly as large as those from Covoli di Velo, Grotta Ladrenizza and Ponte Galeria. In GBOC-HOT, GBOC-GBFM and HFM-GBFM plots, *C. lupus* from Grotta Romanelli and *C. mosbachensis* from Cerè fall outside or within the lower portion of the range of extant *C. lupus italicus* (Fig. 5). The specimen of *C. lupus maximus* from Grotte de Jaurens is the largest among the analyzed sample and falls outside the range of the extant Italian wolf. In GDAB-GBFM and GDAB-GBOC plots (Fig. 5) the fossil samples from Covoli di Velo, Grotta di Ladrenizza, Grotta Romanelli and Ponte Galeria, fall within the smallest extant *C. lupus italicus*, with PF-PG1 placed in an intermediate position between Covoli di Velo and Grotta Ladrenizza (larger), and Grotta Romanelli (smaller).

### Frontal sinuses

**Figure 6:**
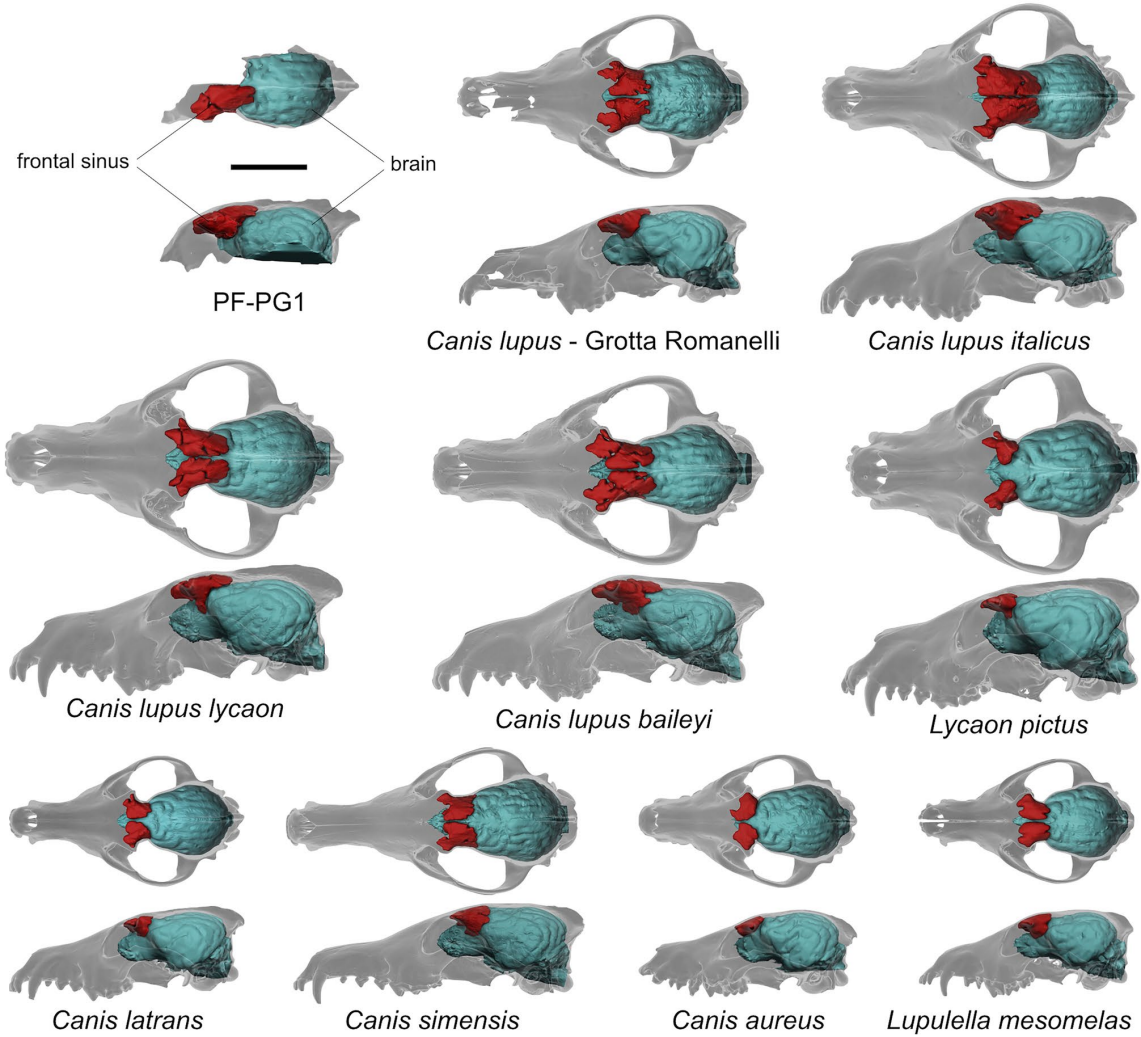
Comparative morphology of the frontal sinuses of PF-PG1 from Ponte Galeria and a selected sample of fossil and extant canids (the complete list of specimens is reported in Supplementary Table S4). Sale bar: 50 mm.

The specimen PF-PG1 shows a well-developed left frontal sinus (Fig. 6) extending from the antorbital constriction up to the fronto-parietal suture. In dorsal view, its anterior portion—between the preorbital constriction and the zygomatic process of the frontal—is laterally expanded, lobed and crossed by a deep transverse groove, while the caudal one—between the zygomatic process of the frontal and the fronto-parietal suture—is narrower, less lobed, with a compact and smooth surface (Fig. 6). In lateral view, the sinus is triangularly shaped and quite domed. The frontal sinus as it appears in Fig. 6, is 43.7 mm long and 19.1 mm wide (the comparative list of measurements is reported in Supplementary Table S4), reaching almost half the length of the brain endocast. In dorsal view, it covers the whole anterior portion of the brain extending to the cruciate sulcus, while laterally it completely covers the orbital gyrus.

The frontal bone of the Ponte Galeria specimen contains a large frontal sinus, morphologically similar to those of fossil (Grotta Romanelli) and extant *C. lupus* (e.g., *C. lupus italicus*, *C. lupus lycaon, C. lupus baileyi*) (Fig. 6). According to Curtis and Van Valkenburgh^37^, the frontal sinuses of *C. lupus* are the largest among all living canids, representing a peculiarity of the species. Frontal sinuses of *L. pictus* are strongly lobed, reduced in size and placed between the preorbital and postorbital constriction. In dorsal view they do not reach the fronto-parietal suture nor the cruciate sulcus of the brain, while in lateral view they do not fully cover the orbital gyrus (Fig. 6). Considering canids of smaller sizes, such as *C. aureus*, *C. latrans* and *Lupulella mesomelas*, their frontal sinuses are strongly reduced with a simplified morphology. They are dorsally flat with a smooth surface, and they never reach the fronto-parietal suture nor the cruciate sulcus of the brain (Fig. 6).

### Brain

**Figure 7:**
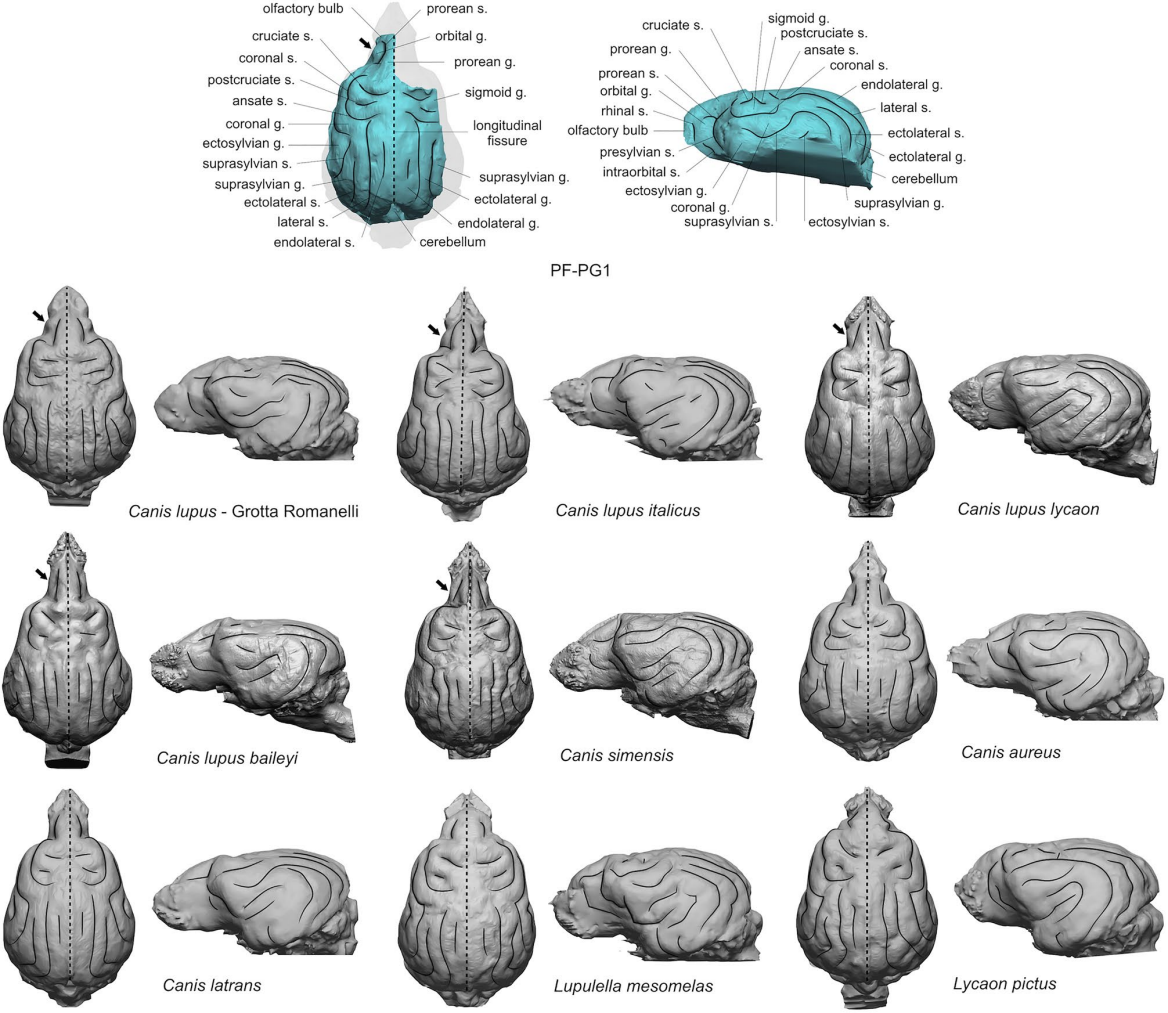
Comparative brain morphology of PF-PG1 specimen from Ponte Galeria and a selected sample of fossil and extant canids (the complete list of specimens is reported in Supplementary Table S5). The black arrows indicate a prominent orbital gyrus distinctive of *Canis lupus* and *C. simensis*. The sample is normalized.

Processing CT-images, we obtained a partial brain endocast (Fig. 7) consisting of an upper left hemisphere with clearly visible convolutions, and a portion of the right one, which shows a smoother surface and less marked sulci. The olfactory bulbs, most of the anterior left hemisphere, the entire basal portion of the cerebrum and almost all the cerebellum are missing. The brain endocast, as it appears in Fig. 7, is 74.1 mm long and 57.2 mm wide (the comparative list of measurements is reported in Supplementary Table S5). In dorsal view, a poorly marked longitudinal fissure divides the telencephalon into two almost symmetrical hemispheres with convolutions represented—from front to back—by the orbital, prorean, lateral, sigmoid, coronal, endolateral, ectolateral, suprasylvian and ectosylvian gyri, including the respective sulci (for the complete list of the brain convolutions see Fig. 7). In dorsal and lateral views, the brain is morphologically similar to the sample from Grotta Romanelli and to those of the extant *C. lupus italicus*, *C. lupus lycaon*, *C. lupus baileyi* and *C. simensis.* In these specimens, the frontal pole cortex is antero-posteriorly elongated, the orbital gyrus is laterally expanded forming a quite developed “bump” bounded by the intraorbital sulcus, the prorean gyrus is long and bilaterally constricted. Moreover, the orbital region is characterized by the presence of three main sulci: the prorean sulcus, the intraorbital sulcus and a third sulcus. According to Lyras and Van der Geer^38^ the third sulcus is considered typical of *C. lupus*, *C. simensis* and *C. rufus*. In the remaining species of our comparative sample the frontal pole is less elongated, and the orbital gyri are delimited in *C. latrans* and *L. pictus* by the prorean and intraorbital sulcui, while in *C. aureus* and *Lupulella mesomelas* only by the prorean sulcus. On the left hemisphere of extant *C. lupus italicus*, *C. lupus lycaon* and *L. pictus* a small dimple is detectable on the anterior portion of the coronal gyrus which is missing in the rest of the sample, including the specimen from Ponte Galeria and the fossil wolf from Grotta Romanelli and *C. lupus baileyi*. This feature was reported by Radinsky^39^ and Lyras^40^ only in *C. lupus*, *Cuon alpinus* and *L. pictus* and according to the authors it seems to be missing in all other canid species. In Grotta Romanelli and Ponte Galeria specimens, the surface irregularities of the brain endocasts do not allow the identification of this character, which however is not recognizable in *C. lupus baileyi* despite the good quality of the brain model. The brain volume of the specimen from Ponte Galeria has not been measured due to the incompleteness of the endocast.

## Discussion

The wolf from Ponte Galeria dated at ca. 407 ka represents the largest cranial remain of a Middle Pleistocene canid known to date in Europe. Due to the fragmentary nature of the specimen, CT-based methods have been used to acquire a wider set of morphological data, including those offered from the brain and frontal sinuses. The sample from Ponte Galeria shows the following wolf-like features: (I) elevated frontal bones, (II) well-developed sagittal and occipital crests, (III) large and domed frontal sinus which extends to the fronto-parietal suture reaching the cruciate sulcus of the brain, (IV) rostral pole of the brain antero-posteriorly elongated with the orbital gyrus laterally expanded. In size, the specimen PF-PG1 is larger than *C. mosbachensis*, matching the values of moderately large individuals of *C. lupus* (Fig. 5), while morphologically it is almost indistinguishable from the extant wolf and it appears more robust compared to *C. mosbachensis* (Supplementary Fig. S3). In the latter species, the frontals are less swollen than in *Canis lupus*, suggesting a lower extension of the frontal sinuses. Strongly pneumatized and dorsoventrally expanded frontals are considered a distinctive trait of the wolf, since this species possesses the largest frontal sinuses among all extant canids^37^. Although frontal sinuses of *C. mosbachensis* have never been formally described, Tedford et al.^12^ indicates that in this species the frontal sinuses are large, but they never reach the fronto-parietal suture—character 34(2) of their phylogenetic analysis—as instead occurs in the wolf—character 34(3). Large sinuses have been found throughout our sample of Late Pleistocene and extant *C. lupus* (Fig. 6), thus confirming the consistency of the character 34(3) scored by Tedford et al.^12^ for *C. lupus*. In the sample of Ponte Galeria, the left frontal sinus is large, domed and extends up to the fronto-parietal suture, perfectly corresponding in size and shape to those of the wolf (Fig. 6).

The neuroanatomy of fossil canids was explored by few authors^4,38–41^ and studies on extensive fossil specimens have never been performed. Additionally, no brain endocasts of Middle Pleistocene wolf-like canids have been documented in Europe. As a result, our knowledge of the brain anatomy of *C. mosbachensis* and early *C. lupus* is limited. The partial brain endocast of PF-PG1, with an age of ca. 407 ka, fills this gap and appears as a brain of an extant wolf. The overall size, the antero-posteriorly elongated frontal pole cortex, the convolution pattern and the presence of a marked orbital gyrus bounded by the three sulci, one of which—the third sulcus, is considered typical of *C. lupus*, *C. simensis* and *C. rufus*^38^, point out the morphological affinities with the wolf (Fig. 7). Studies on the brain anatomy of social carnivorans evidenced a possible relation between the development of the frontal pole cortex and sociality. For instance, in domestic dogs the precruciate and prorean gyri are involved in social action and interaction^42^, the prorean gyrus has been also linked to the emergence of pack structure in canid evolution^43^, and in extant *Crocuta crocuta* the expansion of the frontal cortex has been linked to increased sociality^44^. In PF-PG1 the overall development of the rostral portion of the brain, including the orbital, precruciate and prorean gyri, matches the proportions of *C. lupus* and *C. simensis*, which are both able to form packs spanning from 3 to more than 13 individuals, depending on food availability^45^. Hence the morphological resemblance with PF-PG1 allows to assume that such pack structures were already present in Middle Pleistocene wolves of Europe, although additional paleoneurological studies on the frontal cortex of fossil canids are required to consolidate this hypothesis.

Considering the above, we ascribe the Ponte Galeria specimen to an adult *Canis lupus*. We excluded the attribution to *Canis mosbachensis* because in this hypothesis the specimen from Ponte Galeria would represent the largest and most robust cranial remain of *C. mosbachensis* ever recorded, contradicting what is generally claimed for this species, namely that it is slender and of a size comparable to that of the extant subspecies *C. lupus pallipes*^11^. It could be argued that size alone should not be taken as a valid criterion for specific distinction, especially considering the huge biometric and morphological variability observed between different populations of extant *C. lupus*. The Mosbach wolf has often been considered a chronosubspecies of the wolf, *C. lupus* mosbachensis^23,46^, a view that only after the description of the large sample from the Early Pleistocene of Untermassefeld became less widespread^11,12^ (Supplementary Note 1). Emerging data on dental remains of Middle Pleistocene canids from Mediterranean Europe, further support a close ancestor-descendent relation with *C. lupus* and confirm the difficulties in identifying valuable diagnostic characters for *C. mosbachensis*^4,10,47^. In wide-ranging mammals like *C. lupus* the body size is known to vary according to several factors, for instance reflecting ecomorphological adaptations to different environments or climatic conditions, the availability of trophic resources, and competition with sympatric species. Investigating size fluctuations through time adds a dimension of complexity while not excluding factors that operate across other scales. Increasingly, many studies recognized differences between trends advocated for explaining the general evolution of large mammal species and fluctuations that become evident at a closer geographical scale, as for instance documented in Europe for the spotted hyena^48^, the wild boar^49^ and the horse^50^. As for *C. lupus*, differences in size fluctuations through time were noted even comparing the geographically close fossil records of France and Italy^51^. However, recognizing the complex interplay of these factors in explaining the observed variation in *C. lupus* underlines a striking contrast with the lower dimensional variability of *C. mosbachensis*. Samples assigned to the Mosbach wolf span in chronology about twice the time from the FO of *C. lupus* to the present day, and yet none of them includes outliers of a large size comparable to that of subsequent populations of *C. lupus*^11,19,52^. The problem arises when approaching the “transitional” period of the late Middle Pleistocene, with forms of intermediate size such as *C. lupus lunellensis* that can be hardly significantly separated on a biometrical ground^10,19,22,23^. Although it cannot be completely ruled out that some Middle Pleistocene forms of *C. mosbachensis* may have reached a large body size, a significant preservation bias is unlikely considering the abundance of dental remains in the early Middle Pleistocene^10,19,53^. A potential explanation may rest on the faunal turnover occurred at the time of the FO of *C. lupus*, the Galerian-Aurelian transition, which saw the extinction of several large-sized carnivorans^14^. This renewal may have open new niches for an opportunistic predator to be exploited, hence eventually promoting local adaptations in size in different ecological scenarios.

The Ponte Galeria wolf is dated close to the Mid-Brunhes Event (ca. 424 ka; MIS 12–11 transition), which marks the end of the Early-Middle Pleistocene Transition and the consolidation of the glacial cycles ruled by a 100 kyr periodicity^54^. After this transition an increase in the amplitude of interglacials is recorded with one of the longest and warmest interglacial occurring right after the extreme MIS 12 glacial, during MIS 11^55^. In central Italian Peninsula, MIS 11 seems to have been characterized by a marked seasonality, as suggested by the analysis of the diets of fossil herbivorous ungulates from Fontana Ranuccio (408 ± 10 ka; Anagni, Central Italy)^56^, and by pollen-based climate reconstructions of Combourieu-Nebout et al.^57^, in which the lowest winter temperatures of all the examined Middle Pleistocene interglacials are inferred during MIS 11 (in the Boiano locality; Campobasso, Central Italy). The earliest dispersal of *C. lupus* is one of the bioevents selected to define the beginning of the Aurelian Mammal Age of the Italian large mammal biochronological scale^14^. Two additional taxa appeared during the Aurelian, *Megaloceros giganteus* and *Ursus spelaeus*, but studies of the last decades suggest an older diffusion, occurred during MIS 11 and MIS 13, respectively^29,58^. After Gliozzi et al.^14^, many studies added important information on the timing of dispersals or extinction of several large mammals^59,60^. During MIS 13–10, multiple key bioevents occurred in Europe favoring the spread of *Bos primigenius*^32^, *Equus hydruntinus*^61^ and *Dama clactoniana*^62,63^, evidencing how terrestrial ecosystems were strongly affected by the Mid-Brunhes Event. Considering the large turnover recorded during the Mid-Brunhes Event, it is thus possible that the severe glacial conditions of MIS 12 may have played a role in triggering the spread of large-sized wolves, and/or that the warmer and seasonal conditions started after MIS 12 favored the establishment of viable populations. The presence of morphologically modern wolves in North America in the late Early Pleistocene^12^ and the abundance of *C. mosbachensis* in East Asia during the early Middle Pleistocene^64^ suggest a dispersion of *C. lupus* into Europe, rather than a local origin of a modern phenotype. In this scenario large-sized wolves may have dispersed into Europe encountering or even interbreeding with populations of *C. mosbachensis* previously inhabiting the region. The impoverishment in the carnivoran guild may have favored the spread of these newcomers^14^, as well as allowing them to occupy a wider range of niches compared to *C. mosbachensis*. Considering dental measurements, large-sized wolves are documented since MIS 8-7 in Europe^52,53^, but the Ponte Galeria cranium supports an earlier arrival of *C. lupus* during MIS 11, ca. 400 ka. To better understanding the tempo and mode of the acquirement of the modern phenotype along and before the *C. mosbachensis-C. lupus* transition, and possibly of the origin of the wolf, CT-based studies of Early to Middle Pleistocene wolf-like canids are needed.

Concluding, PF-PG1 attests the presence of modern wolf-like morphologies in Europe at 406.5 ± 2.4 ka, suggesting a climate-induced turnover between the latest Early to early Middle Pleistocene forms of *C. mosbachensis* and those succeeding referable to *C. lupus*. It remains to be clarified whether *C. mosbachensis* represents a reliable taxonomic entity or it should rather be considered an early morphotype of *C. lupus*.

## Material and methods

### Studied material

The studied specimen belongs to the fossil mammal collection of the Museo Universitario di Scienze della Terra (MUST), Sapienza University of Rome, and consists of a fragmented neurocranium (PF-PG1) of a wolf-like canid from the Middle Pleistocene of Ponte Galeria. The sample of CT-scanned crania includes: the Middle Pleistocene specimen PF-PG1 from Ponte Galeria; the Late Pleistocene *Canis lupus* P3580 from Grotta Romanelli (n 1)^4^; extant *Canis lupus italicus* (n 7); *Canis lupus lycaon* (n 1); *Canis lupus baileyi* (n 1); *Lycaon pictus* (n 1); *Canis simensis* (n 1); *Canis latrans* (n 1); *Canis aureus* (n 4); *Lupulella mesomelas* (n 1). Morphological and biometric data collected from the literature include: *Canis mosbachensis* from Cerè^65^ and L’Escale^19^; *C. lupus lunellensis* from Lunel-Viel^19^; *C. lupus* from Grotta Romanelli^4^; *C. lupus* from Covoli di Velo and Gotta Ladrenizza^65^; *Canis lupus maximus* from Jaurens^66^; extant *C. lupus italicus*^65^. According to Driesch^67^, 7 cranial variables have been considered (Supplementary Table S3).

### CT-scanning and digital restoration

Tomographic images of *Canis lupus lycaon*, *Canis lupus baileyi* and *Canis simensis* were downloaded from MorphoSource.org (ark:/87602/m4/M113796, ark:/87602/m4/M114099, ark:/87602/m4/M113802). Tomographic images of the rest of the sample were taken using a Philips Brilliance CT 64-channel scanner at M.G. Vannini Hospital (Rome). The cranial fragments were scanned together in the coronal slice plane from front to back. The slice thickness is 0.67 mm with an interslice space of 0.33 mm. No skulls of *Canis mosbachensis* were available for the examination of the frontal sinuses and the brain. CT image processing and measurements of the frontal sinuses (Supplementary Table S4) and brains (Supplementary Table S5) have been acquired using Mimics 21.0. Each fragment of the studied braincase has been digitally reconnected matching the complementary fragments. The high correspondence of the bone margins and the use of 3D reference skulls of both extant and fossil wolves, allowed to obtain a restored 3D model of the Ponte Galeria specimen. The process of fragment alignment was carried out with ZBrush 4R6. The restored model generated during the current study is available in the MorphoSource repository (ark:/87602/m4/408336).

### Analytical methods

Before CT scan the specimen has been sampled. The volcanoclastic matrix occurring in the internal and external parts of the skull was gently removed from the bone using a thin blade. The sediment was split at the binocular, and volcanic components were picked and mounted as polished and carbon-coated thin section. Texture was observed at the Department of Earth Sciences, Sapienza University of Rome, using the polarized optical microscope and the FEI Quanta 400 electron microscope. EMP analyses of glasses were carried out at the CNR-Istituto di Geologia Ambientale e Geoingegneria di Roma, with a Cameca SX50 electron microprobe equipped with five wavelength dispersive spectrometers (WDS). Quantitative analyses were performed using 15 kV accelerating voltage and 15 nA beam current. As standards we employed metals for Mn and Cr, Jadeite for Na, Wollastonite for Si and Ca, Orthoclase for K, Corundum for Al, Magnetite for Fe, Rutile for Ti, Periclase for Mg, F-apatite for P, phlogopite for F, potassium chloride for Cl, barite for S. Counting times for all elements were 20 s on peak and half time on both backgrounds. Light elements (Na, K) were counted first to prevent loss by volatilization. The PAP correction method was used. Glasses were analyzed using a beam diameter of 15 µm to minimize alkali loss. In order to evaluate the accuracy of the analyses, repeated analyses of three international secondary standards (Kakanui augite, Iceladic Bir-1 and rhyolite RLS glasses from USGS) were made prior to any series of measurements. The mean precision from the standard value was about 1% for SiO2, 2% for Al2O3, 5% for K2O, CaO and FeO, and 8–10% for other elements. Moreover, the analytical precision (2 sigma error) is ≤ 1% for elements in the concentration range > 10 wt.% oxide, 5% for elements in the range 2–10 wt.% oxide and better than 10% for elements in the range 0.5–2 wt.% oxide.

## Supplementary Information

Supplementary Information.
