# Supplementary material for: A Middle Pleistocene wolf from central Italy provides insights on the first occurrence of Canis lupus in Europe
Source: Sci Rep. 2022 Feb 25;12:2882. doi: 10.1038/s41598-022-06812-5 (PMC8881584; doi:10.1038/s41598-022-06812-5)
Supplement: Supplementary file 1 — Supplementary Information. [file 41598_2022_6812_MOESM1_ESM.docx]

**Supplementary information**

**A Middle Pleistocene wolf from central Italy provides insights on the first occurrence of *Canis lupus* in Europe**

**Dawid A. Iurino^1,*^, Beniamino Mecozzi^1,2,*^, Alessio Iannucci^1,2^, Alfio Moscarella^1,2^, Flavia Strani^1^, Fabio Bona^3^, Mario Gaeta^2^, Raffaele Sardella^1,2^**

^1^PaleoFactory, Sapienza Università di Roma, Piazzale Aldo Moro 5, 00185, Rome, Italy;

^2^Dipartimento di Scienze della Terra, Sapienza Università di Roma, Piazzale Aldo Moro 5, 00185, Rome, Italy;

^3^Dipartimento di Scienze della Terra “A. Desio”, Via Mangiagalli 34, 20133, Milano, Italy;

^*^Corresponding author: [dawid.iurino@uniroma1.it](mailto:dawid.iurino@uniroma1.it), beniamino.mecozzi@uniroma1.it

**Supplementary Note 1: the taxonomic history of the Mosbach wolf**

The Mosbach wolf, *Canis mosbachensis* Soergel, 1925, is generally considered the ancestor of the extant wolf, *Canis lupus* Linnaeus, 1758, occupying an intermediate position along the wolf evolutionary lineage (*Canis etruscus* Forsyth Major, 1877 *– Canis mosbachensis – Canis lupus*)^4,11,[1,2]^.
Given the lack of clear-cut morphological criteria useful for identifications to the species level, general confusion persists in paleontological literature, affecting the taxonomical attributions proposed during the last century. For instance, the Mosbach wolf was considered a valid and distinct species, *Canis mosbachensis*^2,13,[3,4]^, an advanced form of *Canis etruscus* (*Canis etruscus mosbachensis*^22,[5]^) or an ancestral form of *Canis lupus* (*Canis lupus mosbachensis*^41, [6,7,8,9,10]^). The consistency of this taxon was problematic since its introduction by Soergel^[11]^. The author described a fragmentary mandible found at the Middle Pleistocene site of Jockgrim (Germany) and ascribed it to *Canis neschersensis*. In doing so, he compared the Jockgrim mandible with a canid sample recovered from the Middle Pleistocene locality of Mosbach (Germany), referred for the first time in a publication as *Canis mosbachensis*. This was enough for making *Canis mosbachensis* Soergel, 1925 available for nomenclatural purposes. Nevertheless, information on this comparative sample, including its name, were communicated to Soergel by his friend Otto Schmidtgen, director of the Naturhistorische Museum Landessammlung für Naturkunde Rheinland-Pfalz (Nhm) of Mainz, where the fossil materials from Mosbach were stored (now Naturhistorisches Museum Mainz). Fossils from Mosbach were first described by von Reichenau^[12]^ who attributed all the canid sample to *Canis neschersensis.* Later, the canid remains from Mosbach were referred at least to two species, *Canis mosbachensis* and *Xenocyon lycaonoides*^[6,7]^, whereas the putative presence of *Cuon priscus* from this site was denied by Schütt^[7]^. Unfortunately, not all the canid sample from Mosbach was properly described and figured^[11,12]^, in fact, considering that the material was initially classified as *Canis neschersensis* it is not clear which specimens were successively ascribed to *Canis mosbachensis* and *Xenocyon lycaonoides*, contributing to the taxonomic confusion. Due to the lack of an established type and because during the 20^th^ century other fossils were recovered from Mosbach, *C. mosbachenensis* remains a vaguely defined taxonomic entity.

For more than 70 years, this species lacked standard and well-defined diagnostic characters, until the new findings and the revision of historical collections added crucial information. One of the most informative works was carried out by Sotnikova^[11]^, who described a large sample from the late Early Pleistocene of Untermassfeld. The author provided an exhaustive morphological description of crania, mandibles, isolated teeth and postcranial elements, setting effectively diagnostic features for this taxon. After the description of the sample from Untermassfeld, other studies have focused on the description of cranial material from several sites: Cerè^20^, Contrada Monticelli^42^, L’Escale^19^ (previously referred to as *Canis etruscus* by Bonifay^22^) and Ostiense^10^. These Middle Pleistocene materials, together with the samples from the late Early Pleistocene of Cueva Victoria^13^, revealed a strong affinity between the Mosbach wolf and the extant wolf, especially in that slender forms of fossil and extant *Canis lupus* show frequently *C. mosbachensis*-like characters^10,11^.

A clear example of how problematic the taxonomic attribution of canids can be, is provided by the case of the remains from Cueva Victoria, initially assigned to *C. etruscus*^[13,14]^ or *C. etruscus etruscus*^[15]^, then to *C. mosbachensis*^[16]^, later to *C. arnensis*^[17]^ and finally to *C. mosbachensis* by Bartolini Lucenti et al.^13^.

One of the aspects involved in the taxonomy of wolf-like canids is size, with a trend of increasing dimensions depicted along their evolutionary history^2,18,19,21,[18]^. This trend encompasses the small specimens of *Canis etruscus* of the Early Pleistocene, medium-sized specimens of *Canis mosbachensis* from the late Early to Middle Pleistocene, medium to large-sized specimens of early forms of *Canis lupus* and culminates with the largest forms recognized during the second part of the Late Pleistocene (*Canis lupus maximus*, *sensu* Brugal and Boudadì-Maligne)^2^. Nevertheless, considering size as a tool for taxonomical attribution could be misleading, since the representatives of the genus *Canis* generally have wide geographical distribution and are adapted to a broad range of habitats and environments. For instance, *Canis lupus* is today widespread throughout the northern hemisphere, with populations that largely differ in several phenotypic features (including size). Several subspecies are accordingly recognized, two in Europe, two (or three) in Asia and five in North America. The extant wolf shows a clinal gradient that roughly adheres to Bergmann’s ecogeographical rule, with southern populations generally characterized by a reduced size^[19,20,21,22]^. Like the extant wolf, *Canis mosbachensis* had a wide geographical distribution and may show geographical differences^47^. Although limited in scope, this hypothesis is supported by some data available in the literature, with the Early to Middle Pleistocene northern samples (e.g., Untermassfeld and Mosbach) including larger individuals than those recovered from southern localities (e.g., Pirro Nord, L’Escale and Petralona)^2,66^.

The canid fossil record is mainly represented by isolated or fragmentary craniodental elements, whereas postcranial bones and nearly complete crania are exceptionally rare. Considering Pleistocene canids, isolated teeth are the most abundant and best preserved remains, which explain why they are often considered for taxonomical studies. In this context, the size of the lower carnassial (M_1_) has been considered a valuable parameter to discriminate *Canis mosbachensis* and *Canis lupus*^2,22^. Nevertheless, a broad biometric comparison of fossil and extant canids demonstrated that there is large overlap in tooth size between *Canis mosbachensis* and early forms of *Canis lupus*^10^. This emphasizes the poor reliability of taxonomic identifications exclusively based on dimensional criteria, especially for those fossils chronologically referred to MIS 12-9, when the *Canis mosbachensis-Canis lupus* transition occurred. Likewise, size-based identification of Villafranchian forms should be avoided, especially those between 1.6 – 1.4 Ma, when the *Canis etruscus–Canis mosbachensis* transition took place.

**Supplementary Note 2: geochemical analysis**

The volcanic material occurring in PF-PG1 cranium is represented by whitish ash and pumice clasts. Observed in thin section (Supplementary Fig. S1), the pumice clast shows an aphyric, highly vesicular and vitrophyric texture (i.e., made up almost exclusively of glass). The colourless glass that forms almost exclusively the pumice clast occurring in PF-PG1 has a relatively constant chemical composition (Supplementary Fig. S2), characterized by the silica oversaturation (SiO_2_ =74 wt% on volatiles free basis). This chemical composition plots in the rhyolite field of the TAS (Total Alkali versus Silica) diagram used for the classification of the volcanic rocks and can be considered a rarity at the regional scale. In particular, the only volcanic materials in the “Campagna Romana” with a composition similar to that of the pumice lapilli occurring in the cranium from Ponte Galeria are those erupted between 414.8 ± 2.2 and 406.5 ± 2.4 ka from the Vico volcano^29,35,36^. In particular, the composition of the glass in the cranium PF-PG1 matches those of glasses occurring in the Vico β pyroclastites (Supplementary Fig. S2).

**Supplementary figures**


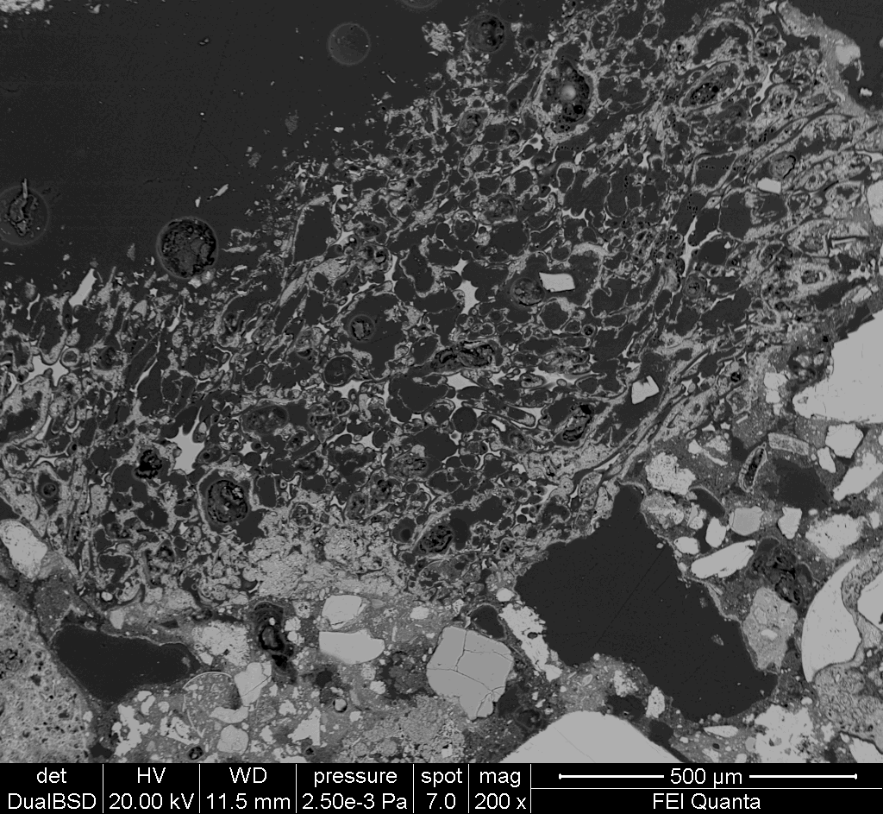


**Supplementary Fig. S1.** Secondary Electron Microscope image of a pumice clast occurring in the PF-PG1 cranium. The pumice clast shows aphyric, highly vesicular (tube-like) and vitrophyric texture.


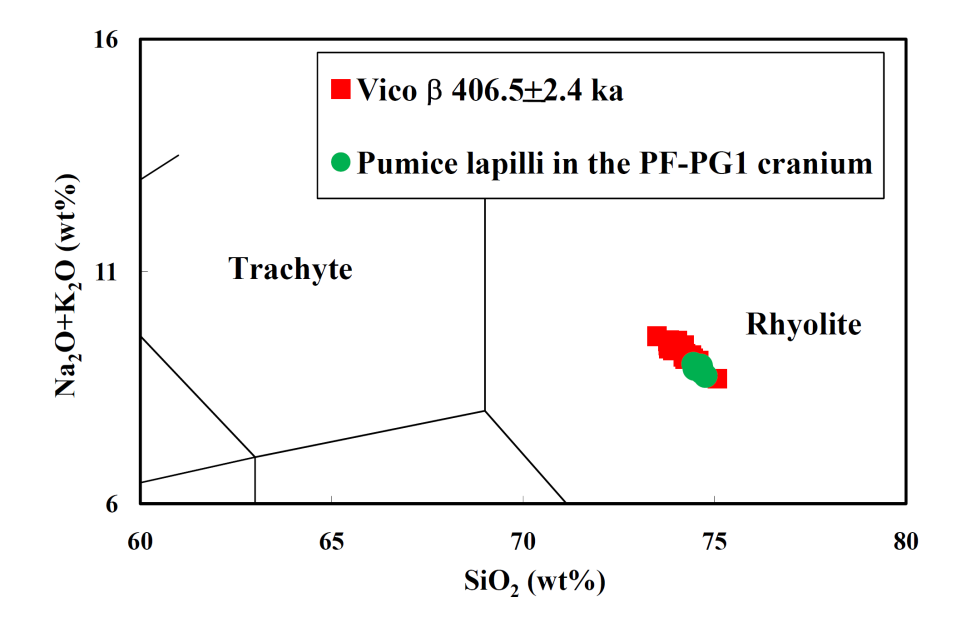


**Supplementary Fig. S2.** Total alkali versus silica (TAS) classification diagram showing the composition of the rhyolitic glass in the PF-PG1 cranium and the match with the composition of glasses in the Vico β pyroclastites (Vico compositions from Pereira et al.^35^).


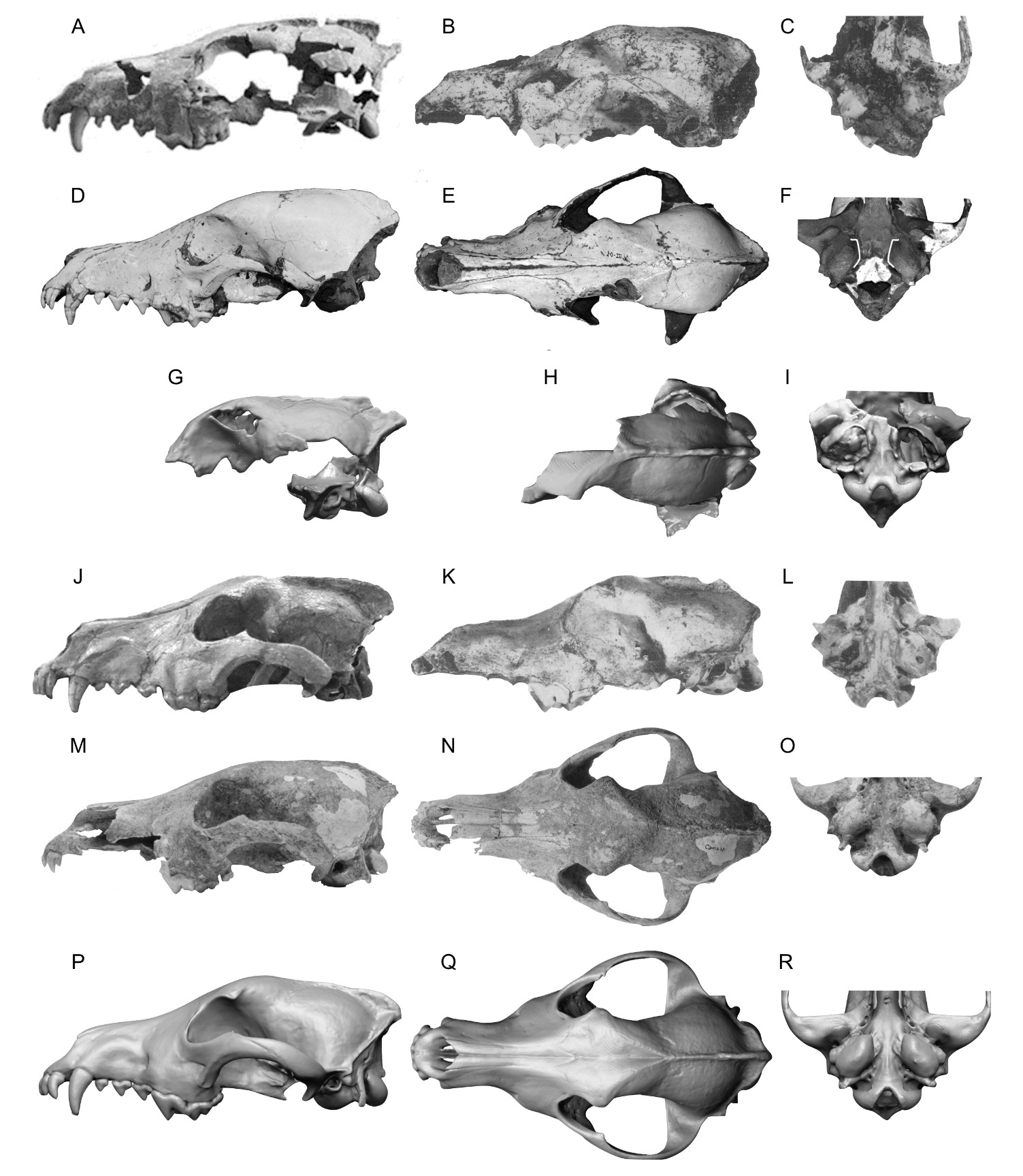


**Supplementary Fig. S3.** Morphological comparison between crania of *Canis mosbachensis* and *Canis lupus*. *C. mosbachensis* from Untermassfeld^40^ in lateral (A) view, *C. mosbachensis* from l’Escale^22^ in lateral (B) and ventral (C) views, *C. mosbachensis* from Cueva Victoria^13^ in lateral (D), dorsal (E) and ventral (F) views, *C. lupus* from Ponte Galeria in lateral (G), dorsal (H) and ventral (I) views, *C. lupus* from La Polledrara di Cecanibbio^26^ in lateral (J) view, *C. lupus lunellensis* from Lunel Viel^22^ in lateral (K) and ventral (L) views, *C. lupus* from Grotta Romanelli^4^ in lateral (M), dorsal (N) and ventral (O) views, extant *C. lupus italicus* in lateral (P), dorsal (Q) and ventral (R) views. The sample is normalized.

**Supplementary tables**

| **Country** | **Species** | **Site** | **MIS** | **References** |
| --- | --- | --- | --- | --- |
| Belgium | *Canis mosbachensis* | Belle-Roche | 13/11 | Ref. 23 |
| Germany | *Canis mosbachensis* | Mosbach 2 | 15/13 | Ref. [11] |
|  | *Canis lupus mosbachensis* | Mauer | 15/13 | Ref. [24] |
|  | *Canis lupus* | Schöningen | 9 | Ref. [25] |
|  | *Canis lupus* | Bilzingsleben II | 11 | Ref. [26] |
|  | *Canis lupus* | Ehringsdorf | 7 | Ref. [26] |
|  | *Canis lupus mosbachensis* | Miesenheim I | 11 | Ref. [27] |
| Poland | *Canis lupus spelaeus* | Biśnik Cave | 8/7 | Ref. [28] |
| Hungary | *Canis mosbachensis* | Tar-köi - Layers 2-3 | 9 | Ref. [29] |
|  | *Canis mosbachensis* | Vertesszöllös | 9 | Ref. [29] |
| Slovakia | *Canis mosbachensis* | Gombaszög | - | Ref. [29] |
| France | *Canis mosbachensis* | L'Escale | 19/16 | Refs 19, 25 |
|  | *Canis mosbachensis* | Château Breccia - 4 | 15/14 | Ref. [30] |
|  | *Canis mosbachensis* | Château Breccia - 2 | 13 | Ref. [30] |
|  | *Canis mosbachensis* | Caune de l'Arago - Ensemble III | 12 | Ref. [31] |
|  | *Canis lupus* | Aldène - Layer K | 12 | Ref. [32] |
|  | *Canis lupus* | Aldène - Layer I | 11 | Ref. [32] |
|  | *Canis lupus lunellensis* | Balaruc VII, | 11/9 | Ref. 19 |
|  | *Canis lupus* | Aldène - Layer G-7-N3b-N4 | 9 | Ref. [32] |
|  | *Canis lupus lunellensis* | Igue des Rameaux | 9 | Refs 2, 19 |
|  | *Canis lupus lunellensis* | Lunel-Viel | 9/7 | Ref. 22 |
|  | *Canis lupus lunellensis* | Abîmes de La Fage | 9/7 | Refs 2, 19 |
|  | *Canis lupus* | Orgnac III | 9/7 | Ref. [33] |
|  | *Canis lupus* | Payre - Level G | 9/7 | Ref. [34] |
|  | *Canis lupus* | Caune de l'Arago - Complexe Superieur | 9/7 | Ref. [31] |
|  | *Canis lupus* | Aldène - Layer D1 and 5 | 8/7 | Ref. [32] |
|  | *Canis lupus lunellensis* | Coudoulous I | 7/6 | Refs 2, 19 |
|  | *Canis* cf. *etruscus* | Grotte XV Vaufrey | 8/6 | Ref. [35] |
| Italy | *Canis mosbachensis* | Grotta di Cerè | - | Ref. 20 |
|  | *Canis lupus* | Malagrotta | 13 | Ref. 34 |
|  | *Canis* cf. *C. mosbachenisis* | Visogliano - Breccia - Lower levels | 12/10 | Ref. [36] |
|  | *Canis mosbachensis* | Ostiense | 11 | Ref. 10 |
|  | *Canis mosbachensis* | Fontana Ranuccio | 11 | Refs 56, [37] |
|  | *Canis lupus* | Castel di Guido | 11 | Ref. [38] |
|  | *Canis* cf*. mosbachensis* | Bristie I | 13/7 | Ref. [39] |
|  | *Canis lupus* | La Polledrara di Cecanibbio | 9 | Refs 26, [40] |
|  | *Canis lupus* | Torre del Pagliaccetto | 9 | Ref. 34 |
|  | *Canis lupus* | Grotta Maggiore di San Bernardino | 8-6 | Ref. [41] |
|  | *Canis lupus* | Quartaccio | 7 | Ref. [42] |
|  | *Canis lupus* | Sedia del Diavolo | 8 | Ref. [43] |
| Spain | *Canis lupus* | Sima de los Huesos | 14 | Ref. [44] |
|  | *Canis lupus mosbachensis* | Ambrona | 11/9 | Ref. [45] |
|  | *Canis lupus mosbachensis* | Ambrona | 7 | Ref. [45] |
|  | *Canis lupus* | Trinchera Dolina - TD10-11 | 11/10 | Ref. [46] |
|  | *Canis mosbachensis* | Grotte de la Carriére | 9 | Ref. [47] |
|  | *Canis lupus* | Trinchera Galeria GII-GIII | 9/8 | Ref. [48] |
|  | *Canis lupus* | Bolomor Cave - II | 8 | Ref. [49] |
|  | *Canis lupus* | Cuesta de la Bajada | 8/7 | Ref. [50] |
|  | *Canis lupus* | Arlanpe | 7/6 | Ref. [51] |
|  | *Canis lupus* | Bolomor Cave - III | 7/5 | Ref. [49] |
| Portugal | *Canis mosbachensis* | Punta Lucero | 15/11 | Ref. [52] |
|  | *Canis lupus* aff*. mosbachensis* | Galeria Pesada | 7 | Ref. [53] |
| Greece | *Canis* sp. | Marathousa 1 | 12/11 | Ref. [54] |
|  | *Canis lupus mosbachensis* | Petralona | 8/7 | Ref. [8] |
| Caucasus | *Canis mosbachensis* | Treugol’naya Cave - Layer 5 | 12/10 | Ref. [55] |
|  | *Canis mosbachensis* | Kudaro 1 - layers 8, 5 | 11 | Ref. [56] |
|  | *Canis mosbachensis* | Treugol’naya Cave - Layer 4 | 9/8 | Ref. [55] |
|  | *Canis lunellensis* | Kudaro 3 - layers 7-6 | 9/8 | Ref. [56] |

**Supplementary Tab. S1.** European record of Middle Pleistocene wolf-like canids of relevance for the *C. mosbachensis*-*C. lupus* transition.

| **Analysis** | **1** | **2** | **3** | **4** | **5** | **6** | **7** |
| --- | --- | --- | --- | --- | --- | --- | --- |
| SiO_2_ | 70.95 | 68.98 | 71.00 | 70.86 | 70.11 | 70.04 | 70.07 |
| TiO_2_ | 0.14 | 0.10 | 0.15 | 0.09 | 0.12 | 0.14 | 0.11 |
| Al_2_O_3_ | 13.23 | 12.84 | 13.31 | 13.29 | 13.23 | 13.18 | 13.20 |
| FeO | 1.11 | 1.02 | 1.05 | 1.07 | 1.06 | 1.07 | 1.08 |
| MnO | 0.11 | 0.12 | 0.09 | 0.07 | 0.06 | 0.15 | 0.07 |
| MgO | 0.05 | 0.08 | 0.07 | 0.07 | 0.06 | 0.07 | 0.07 |
| CaO | 1.00 | 0.99 | 1.02 | 1.04 | 1.06 | 1.01 | 1.02 |
| Na_2_O | 2.69 | 2.67 | 2.69 | 2.56 | 2.77 | 2.70 | 2.68 |
| K_2_O | 5.76 | 5.62 | 5.71 | 5.74 | 5.71 | 5.67 | 5.75 |
| P_2_O_5_ | 0.00 | 0.01 | 0.02 | 0.00 | 0.00 | 0.01 | 0.00 |
| F | 0.42 | 0.38 | 0.48 | 0.51 | 0.54 | 0.64 | 0.49 |
| Cl | 0.24 | 0.26 | 0.20 | 0.24 | 0.23 | 0.23 | 0.25 |
| SO_3_ | 0.00 | 0.02 | 0.02 | 0.00 | 0.00 | 0.02 | 0.03 |
| Total | 95.71 | 93.09 | 95.80 | 95.53 | 94.94 | 94.94 | 94.81 |

**Supplementary Tab. S2.** Electron Microprobe analyses of glass occurring within the PF-PG1 specimen.

| **Species** | **Site** | **Catalo.**  **number** | **References** | **GDAB** | **GMB** | **GBOC** | **GBFM** | **HFM** | **GNB** | **HOT** |
| --- | --- | --- | --- | --- | --- | --- | --- | --- | --- | --- |
| *Canis mosbachesis* | Cerè | V182/2 | Refs 20, 65 | - | - | 37.4 | 19.4 | 13.2 | - | - |
| *Canis mosbachesis* | Cerè | V375/1 | Refs 20, 65 | - | - | 38.5 | 19.8 | 15.8 | - | - |
| *Canis mosbachesis* | Cerè | V375/2 | Refs 20, 65 | - | - | 37.0 | 18.8 | 14.6 | - | - |
| *Canis lupus* | Ponte Galeria | PF-PG1 | This work | 25.1 | 78.4 | 41.6 | 21.1 | 19.5 | 57.0 | 57.6 |
| *Canis lupus* | Covoli di Velo | IG VR83878 | Ref. 65 | 24.0 | 77.0 | 44.0 | 22.0 | 19.0 | 68.2 | 55.3 |
| *Canis lupus* | Grotta di Ladrenizza | MGP 26644 | Ref. 65 | 26.0 | 77.9 | 45.8 | 23.4 | 18.5 | - | 57.2 |
| *Canis lupus* | Grotta Romanelli | P3580 | Ref. 65 | 24.7 | 65.0 | 38.4 | 19.5 | 14.6 | 61.0 | 48.0 |
| *Canis lupus lunellensis* | Lunel-Viel 1 | LVI-9-2206 | Ref. 19 | - | 66.7 | 38.2 | - | - | 61.4 | - |
| *Canis lupus lunellensis* | Lunel-Viel 1 | LVI-9-1031 | Ref. 19 | - | 73.6 | 47.0 | - | - | 61.9 | 53.2 |
| *Canis lupus lunellensis* | Lunel-Viel 1 | LVI-9-20207 | Ref. 19 | - | 66.3 | 39.2 | - | - | 60.0 | 56.0 |
| *Canis lupus maximys* | Grotte de Jaurens | FSL 300422 | Ref. 66 | - | - | 53.4 | 24.0 | 15.1 | - | 65.3 |
| Extant *Canis lupus* | | MSNM 7832 | Ref. 65 | 30.0 | 75.0 | 40.5 | 21.5 | 19.1 | 61.2 | 55.0 |
|  |  | MSNM 7831 |  | 25.5 | 69.0 | 38.3 | 18.8 | 17.0 | 59.0 | 40 |
|  |  | MSNM 6592 |  | 29.6 | 77.0 | 44.3 | 25.0 | 21.4 | 59.0 | - |
|  |  | USP 2006 |  | 31.6 | - | 46.8 | 23.6 | 20.5 | 64.0 | 63.0 |
|  |  | USP 1852 |  | 23.7 | 80.5 | 44.0 | 22.6 | 17.2 | 65.0 | 58.0 |
|  |  | MRSNT 180 |  | 29.6 | 79.7 | 47.0 | 25.0 | 19.6 | 69.0 | 60 |
|  |  | MRSNT 171 |  | 31.8 | 79.0 | 48.2 | 24.5 | 18.5 | - | 62.0 |
|  |  | MRSNT 7854 |  | 29.0 | 72.4 | 40.5 | 21.5 | 18.0 | 62.4 | 58.0 |
|  |  | MRSNT ?? |  | 31.0 | 79.5 | 47.5 | 24.5 | 18.3 | 64.0 | 63.0 |
|  |  | MRSNT 175 |  | 28.0 | 77.5 | 48.2 | 26.2 | 20.6 | 68.0 | 66.0 |
|  |  | MRSNT 179 |  | 29.7 | 83.3 | 46.8 | 25.0 | 19.0 | 67.0 | 68.0 |
|  |  | MRSNT 184 |  | 28.3 | 80.5 | 47.0 | 25.0 | 18.0 | 69.3 | 61.0 |
|  |  | MRSNT 7284 |  | 30.0 | 86.0 | 51.0 | 26.0 | 19.0 | 72.0 | 60.5 |
|  |  | MRSNT 174 |  | 28.0 | 75.4 | 45.0 | 24.3 | 19.0 | 66.0 | 60.0 |
|  |  | MRSNT 4921 |  | 30.0 | 77.0 | - | - | - | 63.7 | - |
|  |  | MRSNTT 608 |  | 31.2 | - | - | - | - | - | - |
|  |  | MRSNTT 606 |  | 30.5 | 76.2 | 42.3 | 21.0 | 17.6 | 66.0 | 53.0 |
|  |  | MSNG 53966 |  | 29.3 | 79.9 | 47.0 | 24.0 | 19.0 | 67.0 | - |
|  |  | MSNG 53965 |  | 27.6 | 73.9 | 40.7 | 22.5 | 18.6 | 65.7 | - |
|  |  | MSNG 48285 |  | 30.3 | - | 44.5 | 22.2 | 18.0 | 64.0 | - |
|  |  | MSNG 52735 |  | 27.0 | 77.0 | 43.0 | 22.0 | 18.0 | 64.0 | - |
|  |  | CE 17830 |  | 27.3 | 77.6 | 47.0 | 23.0 | 18.0 | - | - |
|  |  | MSNG 197 |  | 27.0 | 77.5 | 43.0 | 22.7 | 18.7 | 62.5 | - |
|  |  | DZR 30 |  | 29.7 | 76.0 | 44.0 | 23.0 | 19.3 | 67.7 | 58.0 |
|  |  | DZR 3 |  | 28.4 | 72.3 | 41.0 | 22.0 | 16.3 | 65.0 | 53.6 |
|  |  | DZR 23 |  | 28.2 | - | - | - | - | - | - |
|  |  | DZR 13 |  | 27.0 | 61.0 | 40.7 | 20.0 | 18.6 | 65.0 | 53.0 |
|  |  | DZR 27 |  | 28.0 | 73.0 | 41.3 | 21.8 | 19.4 | 65.4 | 55.7 |
|  |  | DZR 16 |  | 28.2 | 71.7 | 39.0 | 20.3 | 17.0 | 63.7 | 51.0 |
|  |  | DZR 21 |  | 26.0 | 71.8 | 40.5 | 21.2 | 18.3 | 64.0 | 56.0 |
|  |  | DZR 34 |  | 30.0 | 76.4 | 44.0 | 21.6 | 19.5 | 66.0 | 58.1 |
|  |  | DZR 20 |  | 27.0 | 72.2 | 42.5 | 22.6 | 20.0 | 68.0 | 53.0 |
|  |  | DZR 14 |  | 26.6 | 69.0 | 42.0 | 22.6 | 18.2 | 64.7 | 52.0 |
|  |  | DZR 28 |  | 25.7 | 72.0 | 43.0 | 21.0 | 18.0 | 65.7 | 53.0 |
|  |  | DZR 22 |  | 28.6 | 64.0 | 44.6 | 23.0 | 18.5 | 63.3 | 56.0 |
|  |  | DZR 18 |  | 26.0 | 73.0 | 42.7 | 22.0 | 19.3 | 67.6 | 55.4 |
|  |  | DZR 19 |  | 27.5 | 70.4 | 40.3 | 20.2 | 18.8 | 63.0 | 51.0 |
|  |  | DZR 7 |  | 27.2 | 75.0 | 42.3 | 22.0 | 18.0 | 65.0 | 54.0 |
|  |  | DZR 1 |  | 26.2 | 76.0 | 42.9 | 20.6 | 18.3 | 64.7 | 59.0 |
|  |  | DZR 29 |  | 29.2 | 79.0 | 44.3 | 22.0 | 18.4 | 66.0 | 60.0 |
|  |  | DZR 8 |  | 29.8 | 73.6 | 42.0 | 21.3 | 19.5 | 63.0 | - |
|  |  | DZR 9 |  | 26.0 | 62.0 | 41.0 | 20.6 | 19.2 | 65.0 | 55.0 |
|  |  | DZR 25 |  | 27.4 | 71.8 | 39.8 | 20.7 | 17.0 | 63.6 | 52.7 |
|  |  | DZR 10 |  | - | - | - | - | - | 67.0 | - |
|  |  | DZR 2 |  | 27.5 | 76.7 | 45.6 | 22.3 | 17.2 | 66.3 | 58.0 |
|  |  | DZR 6 |  | 27.3 | 73.0 | 39.0 | 20.4 | 18.6 | 62.5 | 53.2 |
|  |  | DZR (no num) |  | 24.4 | 75.0 | 43.0 | - | - | 64.4 | - |
|  |  | DZR 0143CV |  | 27.0 | 68.7 | 39.0 | 20.2 | 16.4 | 60.5 | 53.0 |
|  |  | DZR 0142CV |  | 25.5 | 72.7 | 39.6 | 20.6 | 19.0 | 65.0 | 51.0 |
|  |  | ISPRA 9184 |  | 30.0 | 78.0 | 47.0 | 24.0 | 21.7 | 61.0 | 62.0 |
|  |  | ISPRA 9047 |  | 25.9 | 71.0 | 40.0 | 20.6 | 19.0 | 64.5 | 51.4 |
|  |  | ISPRA 9064 |  | 28.0 | 79.0 | 45.0 | 21.0 | 18.0 | 67.5 | 54.6 |
|  |  | ISPRA 9053 |  | 27.0 | 68.0 | 39.0 | 20.6 | 17.4 | 59.0 | 49.0 |
|  |  | ISPRA 9055 |  | 27.3 | 67.0 | 38.3 | 20.2 | 16.6 | 63.0 | 50.0 |
|  |  | ISPRA 9058 |  | 29.0 | 74.0 | 43.0 | 23.5 | 21.0 | 59.0 | 55.0 |
|  |  | ISPRA 9070 |  | 25.4 | 70.0 | 39.4 | 19.0 | 18.0 | 58.0 | 54.0 |
|  |  | ISPRA 9049 |  | 30.0 | 71.0 | 41.3 | 20.6 | 18.3 | 62.0 | 53.0 |
|  |  | ISPRA 9057 |  | 29.5 | 75.5 | 39.0 | 19.0 | 18.0 | 60.0 | 56.4 |
|  |  | ISPRA 9190 |  | 27.0 | 74.0 | 41.8 | 21.0 | 18.4 | 62.0 | 60.0 |
|  |  | ISPRA 9076 |  | 28.7 | 70.7 | 39.7 | 21.0 | 17.5 | 60.0 | 54.2 |
|  |  | ISPRA 9060 |  | 26.3 | 74.2 | 45.0 | 23.0 | 21.3 | 63.5 | 60.2 |
|  |  | ISPRA 7771 |  | 28.0 | 74.0 | 42.4 | 20.0 | 19.0 | 65.0 | 56.0 |
|  |  | ISPRA 9069 |  | 27.0 | 73.4 | 41.0 | 21.0 | 18.7 | 62.0 | 56.4 |
|  |  | ISPRA 9087 |  | 27.0 | 71.0 | 44.2 | 22.0 | 19.5 | 62.0 | 55.0 |
|  |  | ISPRA 9063 |  | 28.0 | 73.4 | 42.0 | 22.4 | 18.0 | 65.0 | 56.4 |
|  |  | ISPRA 9185 |  | 28.0 | 71.0 | 40.0 | 20.6 | 17.0 | 64.0 | 51.4 |
|  |  | ISPRA 9052 |  | 27.0 | 75.0 | 39.0 | 21.7 | 18.3 | 61.5 | 54.3 |
|  |  | ISPRA 9189 |  | 27.0 | 73.0 | 40.3 | 20.6 | 19.0 | 65.0 | 54.0 |
|  |  | ISPRA 9056 |  | 25.0 | 71.0 | 38.0 | 19.2 | 17.0 | 63.0 | 52.7 |
|  |  | ISPRA 9065 |  | 27.2 | 73.0 | 41.7 | 20.6 | 18.3 | 63.0 | 54.0 |
|  |  | ISPRA 9068 |  | 28.0 | 71.0 | 40.6 | 21.6 | 17.4 | 62.0 | 51.6 |
|  |  | ISPRA 9054 |  | 27.3 | 69.0 | 44.3 | 20.7 | 18.0 | 60.0 | 53.0 |
|  |  | ISPRA 7772 |  | 27.6 | 78.6 | 42.3 | 20.7 | 18.0 | 63.7 | 59.0 |
|  |  | ISPRA 9059 |  | 26.0 | 69.5 | 40.3 | 23.2 | 18.0 | 64.0 | 54.0 |
|  |  | ISPRA 9100 |  | 25.3 | - | - | - | - | 65.0 | - |
|  |  | ISPRA 9088 |  | 28.0 | 71.4 | 43.7 | 23.1 | 17.7 | 65.5 | 53.0 |
|  |  | ISPRA 9618 |  | 31.0 | 82.0 | 44.0 | 23.0 | 21.0 | 66.6 | 59.0 |
|  |  | ISPRA 7945 |  | 31.0 | 80.0 | 46.0 | 23.4 | 19.7 | 64.4 | 60.0 |
|  |  | ISPRA 9607 |  | 25.5 | 76.0 | 44.0 | 21.0 | 16.0 | 63.0 | 55.0 |
|  |  | ISPRA 7939 |  | 27.6 | 71.0 | 39.0 | 20.4 | 16.3 | 61.8 | 53.6 |
|  |  | ISPRA 9620 |  | 25.0 | 71.0 | 41.5 | 21.4 | 17.0 | 63.6 | 53.0 |
|  |  | ISPRA 7770 |  | 28.0 | 78.0 | 43.3 | 21.6 | 17.5 | 64.0 | 59.0 |
|  |  | ISPRA 9182 |  | 27.0 | 77.6 | 42.0 | 23.0 | 20.0 | 66.0 | 59.0 |
|  |  | ISPRA 9085 |  | 27.0 | 67.5 | 38.0 | 19.0 | 16.0 | 62.0 | 50.0 |
|  |  | ISPRA 7775 |  | 27.0 | 72.0 | 42.5 | 21.0 | 18.0 | 65.0 | 53.0 |
|  |  | ISPRA 9608 |  | 29.0 | 76.0 | 46.3 | 22.0 | 17.6 | 65.5 | 54.7 |
|  |  | ISPRA 7946 |  | 28.0 | 73.0 | 41.3 | 20.0 | 19.0 | 66.0 | 57.0 |
|  |  | ISPRA 7277 |  | 28.2 | 73.0 | 44.0 | 23.0 | 19.0 | 67.0 | 55.0 |
|  |  | ISPRA 7284 |  | 27.4 | 71.7 | 39.4 | 20.3 | 17.7 | 65.4 | 53.0 |
|  |  | ISPRA 4399 |  | 25.8 | 71.7 | 43.2 | 23.0 | 18.0 | 66.4 | 53.0 |
|  |  | ISPRA 885 |  | 26.4 | 69.0 | 42.0 | 22.3 | 17.0 | 62.7 | 52.0 |
|  |  | ISPRA 6883 |  | 27.0 | 68.6 | 40.0 | 21.0 | 17.0 | 65.0 | 54.0 |
|  |  | ISPRA 9617 |  | 27.3 | 69.0 | 39.0 | 21.0 | 16.0 | 63.0 | 51.0 |
|  |  | ISPRA 9605 |  | 27.0 | 73.0 | 40.8 | 21.7 | 18.0 | 64.0 | 53.0 |
|  |  | ISPRA 9112 |  | 28.0 | 70.5 | 40.0 | 20.0 | 17.0 | 61.6 | 50.0 |
|  |  | ISPRA 9048 |  | 29.0 | 73.2 | 40.0 | 21.2 | 18.0 | 65.7 | 55.0 |
|  |  | ISPRA 7287 |  | 25.4 | 67.7 | 39.2 | 20.5 | 16.0 | 62.5 | 49.0 |
|  |  | ISPRA 45? |  | 26.4 | 75.0 | 40.7 | 21.0 | 17.3 | 68.0 | 60.5 |
|  |  | ISPRA 9110 |  | 26.4 | 77.0 | 43.4 | 21.3 | 18.0 | 63.4 | 61.4 |
|  |  | ISPRA 6742 |  | 27.7 | 75.6 | 44.0 | 21.4 | 16.2 | 61.0 | 55.0 |
|  |  | ISPRA 9111 |  | 28.0 | 75.0 | 43.0 | 22.0 | 17.0 | 64.5 | 55.0 |
|  |  | ISPRA 6743 |  | 30.4 | 72.3 | 43.4 | 22.0 | 17.5 | 65.2 | 57.0 |
|  |  | ISPRA 9191 |  | 29.0 | 73.0 | - | - | - | 62.0 | - |
|  |  | ISPRA 7292 |  | 26.6 | 74.0 | - | - | - | 64.7 | 55.6 |
|  |  | ISPRA 9117 |  | 29.0 | 75.0 | 43.2 | 22.7 | 17.0 | 64.0 | 56.2 |
|  |  | ISPRA 6898 |  | 30.0 | 73.0 | 40.0 | 21.0 | 17.0 | 62.4 | 58.0 |
|  |  | ISPRA 6884 |  | 25.0 | 68.4 | 41.0 | 20.6 | 16.0 | 63.0 | 55.0 |
|  |  | ISPRA 9142 |  | 27.2 | 79.0 | 45.2 | 21.4 | 16.4 | 67.6 | 57.4 |
|  |  | ISPRA 9619 |  | 25.0 | 78.5 | 40.0 | 20.8 | 18.0 | 65.0 | 57.4 |
|  |  | ISPRA 9051 |  | 31.0 | 73.7 | 42.0 | 22.4 | 18.0 | 66.0 | 53.0 |
|  |  | ISPRA 9119 |  | 23.0 | 67.0 | 40.0 | 20.0 | 16.0 | 63.4 | 51.0 |
|  |  | ISPRA 8508 |  | 26.3 | 81.0 | 45.7 | 22.0 | 18.5 | 68.3 | 59.0 |
|  |  | ISPRA 9121 |  | 28.2 | 74.0 | 40.0 | 22.3 | 20.0 | 66.0 | 56.0 |
|  |  | ISPRA 886 |  | 29.0 | 77.0 | 45.0 | 20.5 | 19.0 | 67.0 | 60.0 |
|  |  | ISPRA 7279 |  | 30.5 | 71.3 | 42.0 | 22.0 | 17.5 | 65.0 | 53.0 |
|  |  | ISPRA 7031 |  | 30.0 | 77.5 | 43.0 | 21.0 | 17.0 | 67.0 | 57.0 |
|  |  | ISPRA 5897 |  | 26.6 | 67.7 | 39.0 | 21.0 | 16.0 | 60.0 | 52.0 |
|  |  | ISPRA 5899 |  | 28.0 | 78.4 | 46.6 | 24.0 | 20.0 | 66.8 | 62.0 |
|  |  | ISPRA 6896 |  | 23.2 | 73.5 | 41.0 | 21.0 | 17.0 | 64.0 | 56.0 |
|  |  | ISPRA 9094 |  | 25.0 | 67.3 | 38.0 | 19.7 | 16.0 | 60.5 | 51.0 |
|  |  | ISPRA 4847 |  | 26.0 | 75.0 | 46.0 | 23.0 | 17.4 | 65.0 | 60.0 |
|  |  | ISPRA 9132 |  | 28.0 | 73.0 | 41.6 | 20.5 | 17.7 | 65.5 | 58.0 |
|  |  | ISPRA 6735 |  | 28.4 | 73.0 | 41.0 | 20.5 | 18.5 | 63.2 | 56.0 |
|  |  | ISPRA 5579 |  | 28.4 | 77.7 | 45.0 | 22.0 | 18.0 | 63.7 | 60.0 |
|  |  | ISPRA 7291 |  | 27.5 | 70.6 | 39.0 | 19.3 | 18.0 | 62.5 | 57.0 |
|  |  | ISPRA 6882 |  | 26.0 | 70.0 | 39.4 | 18.7 | 17.0 | 65.0 | 53.6 |
|  |  | ISPRA 7943 |  | 25.0 | 77.4 | 43.6 | 21.3 | 17.6 | 65.4 | 60.0 |
|  |  | ISPRA 3900 |  | 25.2 | 73.5 | - | - | - | 67.3 | - |
|  |  | ISPRA 3899 |  | 26.0 | 70.4 | 44.0 | 23.0 | 18.0 | 63.0 | 51.0 |
|  |  | ISPRA 3902 |  | 28.0 | 77.0 | 43.0 | 21.0 | 17.3 | 65.0 | 59.0 |
|  |  | ISPRA 901 |  | 27.7 | 74.3 | 42.6 | 23.3 | 18.4 | 62.0 | 53.0 |
|  |  | ISPRA 3904 |  | 28.0 | 71.4 | 41.3 | 21.0 | 16.0 | 65.6 | 50.2 |
|  |  | ISPRA 3903 |  | 28.0 | 78.3 | 44.2 | 23.7 | 17.4 | 69.0 | 59.0 |

**Supplementary Tab. S3.** Measurements (mm) of Middle Pleistocene wolf-like canids considered in the study. Abbreviations: **GDAB** - greatest diameter of the auditory bulla, **GMB** - greatest mastoid breadth, **GBOC** - greatest breadth of the occipital condyles, **GBFM** - greatest breadth of the foramen magnum, **HFM** - height of the foramen magnum, **GNB** - greatest neurocranial breadth, **HOT** - height of the occipital triangle.

| **Species** | **Catalo. number** | **L. Sinus length** | **R. Sinus** **length** | **Frontal Sinuses breadth** | **L. Sinus**  **breadth** | **R. Sinus breadth** | **LsW/Fl Ratio** |
| --- | --- | --- | --- | --- | --- | --- | --- |
| *Canis aureus* | MC140 | 25.6 | 22.4 | 33.3 | 15.4 | 15.7 | 0.6 |
| *Canis. aureus* | MC142 | 18.0 | 15.2 | 30.8 | 13.8 | 12.7 | 0.8 |
| *Canis aureus* | MC368 | 25.4 | 26.2 | 39.0 | 18.2 | 19.1 | 0.7 |
| *Canis aureus* | 5366 | 22.9 | 23.1 | 33.8 | 16.0 | 15.5 | 0.6 |
| *Canis latrans* | MC432 | 19.9 | 20.9 | 35.5 | 18.0 | 16.6 | 0.9 |
| *Lupulella mesomelas* | MC61 | 19.3 | 19.7 | 37.2 | 17.4 | 17.5 | 0.8 |
| *Lycaon pictus* | MC438 | 22.6 | 24.1 | 47.8 | 15.1 | 19.1 | 0.6 |
| *Canis simensis* | AMNH 81001 | 29.3 | 28.4 | 38.8 | 19.1 | 18.6 | 0.7 |
| *Canis lupus italicus* | 1 | 57.5 | 53.2 | 61.0 | 29.5 | 30.4 | 0.5 |
| *Canis lupus italicus* | 9 | 48.1 | 49.2 | 58.1 | 27.3 | 26.6 | 0.6 |
| *Canis lupus italicus* | 10 | 52.3 | 53.0 | 57.6 | 27.2 | 28.5 | 0.5 |
| *Canis lupus italicus* | 19 | 47.4 | 45.2 | 52.7 | 25.7 | 25.2 | 0.5 |
| *Canis lupus italicus* | 20 | 53.2 | 54.0 | 53.0 | 25.4 | 25.7 | 0.5 |
| *Canis lupus italicus* | 29 | 56.4 | 52.9 | 63.2 | 29.4 | 31.8 | 0.5 |
| *Canis lupus italicus* | MC481 | 46.5 | 46.5 | 53.7 | 24.1 | 24.8 | 0.5 |
| *Canis lupus lycaon* | TMM M-1701 | 41.3 | 44.5 | 51.5 | 22.9 | 23.9 | 0.5 |
| *Canis. lupus baileyi* | USNM 98037 | 49.6 | 47.2 | 54.8 | 20.4 | 20.9 | 0.4 |
| *Canis lupus*  Grotta Romanelli | P3580 | 37.7 | 37.3 | 47.9 | 23.2 | 24.1 | 0.6 |
| *Canis lupus*  Ponte Galeria | PF-PG1 | 43.8 | - | - | 19.2 | - | 0.4 |

**Supplementary Tab. S4.** Measurements (mm) of the frontal sinuses of the studied sample.

| **Species** | **Catalogue number** | **Telencephalon lenght** | **Telencephalon breadth** |
| --- | --- | --- | --- |
| *Canis aureus* | MC140 | 54.3 | 43.7 |
| *Canis aureus* | MC142 | 60.4 | 44.7 |
| *Canis aureus* | MC368 | 61.6 | 44.0 |
| *Canis aureus* | 5366 | 55.2 | 42.9 |
| *Canis latrans* | MC432 | 64.5 | 42.5 |
| *Lupulella mesomelas* | MC61 | 62.1 | 42.4 |
| *Lycaon pictus* | MC438 | 74.5 | 54.7 |
| *Canis simensis* | AMNH 81001 | 78.4 | 52.0 |
| *Canis lupus italicus* | 1 | 86.6 | 58.8 |
| *Canis lupus italicus* | 9 | 78.3 | 56.1 |
| *Canis lupus italicus* | 10 | 85.5 | 55.3 |
| *Canis lupus italicus* | 19 | 84.7 | 55.3 |
| *Canis lupus italicus* | 20 | 84.3 | 57.9 |
| *Canis lupus italicus* | 29 | 93.1 | 61.0 |
| *Canis lupus italicus* | MC481 | 84.0 | 60.3 |
| *Canis lupus lycaon* | TMM M-1701 | 90.6 | 62.2 |
| *Canis lupus baileyi* | USNM 98037 | 87.7 | 54.5 |
| *Canis lupus*  Grotta Romanelli | P3580 | 77.5 | 53.4 |
| *Canis* *lupus*  Ponte Galeria | PF-PG1 | 72.2 | 57.3 |

**Supplementary Tab. S5.** Measurements (mm) of the brain of the studied sample.

**Supplementary References**

[1] Torre, D. I cani villafranchiani della Toscana. *Palaeontographia Italica*. **63**, 113-138 (1967).

[2] Sotnikova, M. & Rook, L., Dispersal of the Canini (Mammalia, Canidae: Caninae) across Eurasia during the late Miocene to early Pleistocene. *Quat. Int.* **212**(2), 86-97 (2010).

[3] Moyà, J. P. El *Canis etruscus* Major (Carnivora, Mammalia) del Villafranquiense terminal de la Cueva Victoria (Murcia, España). *Endins: publicació d'espeleologia*. pp.43-46 (1981).

[4] Argant, A. Carnivores quaternaires de Bourgogne. *Documents des Laboratoires de Géologie de Lyon*. **115**, 1-301 (1991).

[5] Agustí, J., Moyà-Solà, S. & Pons-Moyà, J. Venta Micena (Guadix Baza basin, South Eastern Spain): its place in the Plio-Pleistocene Mammal succession in Europe. *Geol. Romana.* **25**, 33-62 (1986).

[6] Adam, K. D. Mittelpleistozäne Caniden aus dem Heppenloch bei Gutenberg (Württemberg). *Stuttgart Beitrage Naturkunden*. **27**, 1-46 (1959).

[7] Schütt G. Die Carnivoren von Würzburg- Schalksberg: mit einem Beitrag zur biostratigraphischen und zoogeographischen Stellung der altpleistozanen Wirbeltierfaunen von Mittelmain (Unterfranken). *Neues Jahrbuch der Geologie und Palaöntologie Abhandlungen*. **147**, 61-90 (1974).

[8] Kurtén, B. & Poulianos, A. N. New stratigraphic and faunal material from Petralona Cave with special reference to the Carnivora. *Anthropos*. **4**(1-2), 47-130 (1977).

[9] Pons-Moyà, J. Los carnivoros (Mammalia) de Venta Micena (Granada, España). Paleontologia Evolucìo. *Memoria Especial*. **1**, 109-128 (1987).

[10] Alcalá, L. & Morales, J. Los carnívoros del Pleistoceno medio de Cúllar de Baza-1 y Huéscar-1 (Cuenca de Guadix-Baza). *Trabajos sobre el Neógeno-Cuaternario*. **11**, 215-223 (1989).

[11] Soergel, W. Die Säugetierfauna des altdiluvialen Tonlagers von Jockgrim in der Pfalz. *Zeitschrift der deutschen geologischen Gesellschaft*. **77**, 405-438 (1925).

[12] von Reichenau, W. Beiträge zur näheren Kenntnis der Carnivoren aus den Sanden von Mauer und Mosbach. Bergsträsser. (1906).

[13] Pons-Moyà, J. & Moyà-Solà, S. La fauna de Carnívoros del Pleistoceno medio (Mindel) de la Cueva Victoria (Cartagena, España). *Acta Geol. Hisp*. **13**, 54-58 (1978).

[14] Pons-Moyà, J. El *Canis etruscus* Major (Carnivora, Mammalia) del Villafranquiense terminal de la Cueva Victoria (Murcia, España). *Endins*. **8**, 43-46 (1981).

[15] Gibert Clols, J., Gibert Beotas, L., Fernández Canyadell, C., Robot, F., Iglesias, A. & Gibert Beotas, P. Cueva Victoria: geología, paleontología, restos humanos y edades. *Mem. Arqueol. Reg. Murcia*. **14**, 37-62 (2006).

[16] Martínez-Navarro, B., Belmaker, M. & Bar-Yosef, O. The large carnivores from ‘Ubeidiya (early Pleistocene, Israel): biochronological and biogeographical implications. *J. Hum. Evol*. **56**, 514-524 (2009).

[17] Boudadi-Maligne, M. Canid remains from Cueva Victoria. Specific attribution and biochronological implications. *Mastia.* **11-13**, 393-399 (2014).

[18] Cherin, M., Bertè, D.F., Rook, L. & Sardella, R. Re-defining *Canis etruscus* (Canidae, Mammalia): a new look into the evolutionary history of Early Pleistocene dogs resulting from the outstanding fossil record from Pantalla (Italy). *J. Mamm. Evol*. **21**, 95-110 (2014).

[19] Meiri, S., & Dayan, T. On the validity of Bergmann's rule. *J. Biogeogr*. **30**(3), 331-351 (2003).

[20] Sillero-Zubiri, C., Hoffmann, M. & Macdonald, D. W. eds. Canids: foxes, wolves, jackals, and dogs: status survey and conservation action plan. Gland, Switzerland: IUCN. (2004).

[21] Meiri, S., Dayan, T. & Simberloff, D. Carnivores, biases and Bergmann's rule. *Biol. J. Linn. Soc*. **81**(4), 579-588 (2004).

[22] Meachen, J. A. & Samuels, J. X. Evolution in coyotes (*Canis latrans*) in response to the megafaunal extinctions. *PNAS*. **109**(11), 4191-4196 (2012).

[23] Rixhon, G., Bourlès, D.L., Braucher, R., Siame, L., Cordy, J.M. & Demoulin, A. ^10^Be dating of the Main Terrace level in the Amblève valley (Ardennes, Belgium): new age constraint on the archaeological and palaeontological filling of the Belle-Roche palaeokarst. *Boreas*. **43**(2), 528-542 (2014).

[24] Wagner, G. A., Maul, L. C., Löscher, M. & Schreiber, H. D. Mauer–the type site of *Homo* *heidelbergensis*: palaeoenvironment and age. *Quat. Sci. Rev.* **30**(11-12), 1464-1473 (2011).

[25] Van Kolfschoten, T., Buhrs, E. & Verheijen, I. The larger mammal fauna from the Lower Paleolithic Schöningen Spear site and its contribution to hominin subsistence. *J. Hum. Evol*. **89**, 138-153 (2015).

[26] Heinrich, W.-D. & Maul, L. C. Mortality profiles of *Castor* and *Trogontherium* (Mammalia: Rodentia, Castoridae), with notes on the site formation of the Mid-Pleistocene hominin locality Bilzingsleben II (Thuringia, Central Germany). *Fossil Imprint*. **76**(1), 40–58 (2020).

[27] Kolfschoten, T. & Turner, E. *Early Middle Pleistocene mammalian faunas from Kärlich and Miesenheim I and their biostratigraphical implications*. In (Ed. Turner, C.). The Early Middle Pleistocene in Europe, pp. 227-253. Rotterdam: Balkema. (1996).

[28] Marciszak, A., Socha, P., Nadachowski, A. & Stefaniak, K. Carnivores from Biśnik Cave. *Quaternaire, Hors-série*. **4**, 101-106 (2011).

[29] Jánossy, D. Pleistocene vertebrate faunas of Hungary. (Ed. Akadémiai Kiadó, & Hague). Elsevier, pp 208 (1986).

[30] Argant, A., Argant, J., Jeannet, M. & Erbajeva, M. The big cats of the fossil site Château Breccia Northern Section (Saône-et-Loire, Burgundy, France): stratigraphy, palaeoenvironment, ethology and biochronological dating. *Courier Forschungsinstitut Senckenberg*. **259**, 121-140 (2007).

[31] Moigne, A.M., Palombo, M.R., Belda, V., Heriech-Briki, D., Kacimi, S., Lacombat, F., de Lumley, M.A., Moutoussamy, J., Rivals, F., Quilès, J. & Testu, A. Les faunes de grands mammifères de la Caune de l'Arago (Tautavel) dans le cadre biochronologique des faunes du Pléistocène moyen italien. *L'anthropologie.* **110**(5), 788-831 (2006).

[32] Rossoni-Notter, E., Notter, O., Simone, S. & Simon, P. Acheulean technical behaviors in Aldène cave (Cesseras, Hérault, France). *Quat. Int*. **409**, 149-173 (2016).

[33] Moncel, M. H., Moigne, A. M. & Combier, J. Pre-Neandertal behaviour during isotopic stage 9 and the beginning of stage 8. New data concerning fauna and lithics in the different occupation levels of Orgnac 3 (Ardèche, South-East France): occupation types. *J. Archaeol. Sci*. **32**(9), 1283-1301 (2005).

[34] Ecker, M., Bocherens, H., Julien, M. A., Rivals, F., Raynal, J.P. & Moncel, M. H. Middle Pleistocene ecology and Neanderthal subsistence: insights from stable isotope analyses in Payre (Ardèche, southeastern France). *J. Hum. Evol*. **65**(4), 363-373 (2013).

[35] Guadelli, J. *L. Étude d'une grotte à Ours du Pléistocène moyen en Dordogne: la Grotte XIV*. In Symposium 2: Archaeology and Paleontology in Caves. Proceedings of the 12th International Congress of Speleology, Natural History Museum, Geneva, Swiss Speleological Society (SSS/SGH). **3**, 117-120 (1997).

[36] Abbazzi, L., Fanfani, F., Ferretti, M. P., Rook, L., Cattani, L., Masini, F., Mallegni, F., Negrino, F. & Tozzi, C. New human remains of archaic *Homo sapiens* and lower palaeolithic industries from Visogliano (Duino Aurisina, Trieste, Italy). *J. Archaeol. Sci*. **27**(12),1173-1186 (2000).

[37] Segre, A. & Ascenzi, A. Fontana Ranuccio: Italy's earliest middle Pleistocene hominid site. *Curr. Anthropol.* **25**(2), 230-233 (1984).

[38] Sala, B. & Barbi, G. Descrizione della fauna. Gli scavi a Castel di Guido, il più antico giacimento di cacciatori del Paleolitico inferiore nell’Agro Romano. (Ed. Radmilli, A. M. & Boschian G.). Gli scavi a Castel di Guido. 55-91(1996). Pisa.

[39] Lugli, C. & Sala, B. La teriofauna del Pleistocene medio di Bristie I° (Carso Triestino). *Atti del Museo Civico di Storia Naturale di Trieste*. **48**, 35-58 (2000).

[40] Anzidei, A. P., Angelelli, L., Arnoldus Huizendveld, A., Caloi, L., Palombo, M. R. & Segre, G. Le gisement pléistocène de La Polledrara di Cecanibbio (Rome, Italie). *L’Anthropologie*. **93**, 749-781 (1989).

[41] Cassoli, P. F. & Tagliacozzo, A. I resti ossei di macromammiferi, uccelli e pesci della Grotta Maggiore di San Bernardino sui Colli Berici (VI): considerazioni paleoeconomiche, paleoecologiche e cronologiche. *Bull. Paletnol. Ital*. **85**(1), 1-71 (1994).

[42] Caloi, L., Palombo, M. R. & Zarlenga, F. Late Middle Pleistocene mammal faunas of the Rome area (Bassa Campagna Romana), *Abstract SEQS Symposium ‘Quaternary Stratigraphy in volcanic areas’. Rome, 20—22 September* (1993).

[43] Caloi L., Palombo M. R. Petronio C. 1980. La fauna quaternaria di Sedia del Diavolo (Roma). *Quaternaria*. **22**,177-209 (1993).

[44] Garcıa, N., Arsuaga, J. L. & Torres, T. D. The carnivore remains from the Sima de los Huesos Middle Pleistocene site (Sierra de Atapuerca, Spain). *J. Hum. Evol*. **33**(2-3), 155-174 (1997).

[45] Pérez-González, A., Santonja Gómez, M., Soto, E., Sesé, C., Ruiz Zapata, B., Mora, R., Villa, P., Aleixandre, T., Gallardo, J. & Benito Calvo, A. *The Archaeo-Palaeontological sites of the Middle Pleistocene at Ambrona and Torralba (Soria)* in The geological and paleontological heritage of Central and Eastern Iberia (Iberian Range, Spain), III International Symposium ProGeo on the Conservation of the Geological Heritage, Field Guide. Seminario de Paleontologia de Zaragoza, Madrid. 7–42 (1999).

[46] Rodríguez-Gómez, G., Rodríguez, J., Martín-González, J. A. & Mateos, A. Carnivores and humans during the Early and Middle Pleistocene at Sierra de Atapuerca. *Quat. Int*. **433**, 402-414 (2017).

[47] Prat-Vericat, M., Rufi, I., Llenas, M. & Madurell-Malapeira, J. Middle Pleistocene *Ursus deningeri* from Grotte de la Carrière (Réseau Lachambre, Têt Valley, Eastern Pyrenees). *J. Iber. Geol.* **46**(2), 163-175 (2020).

[48] García, N. & Arsuaga, J. L. The carnivore remains from the hominid-bearing Trinchera-Galería, Sierra de Atapuerca, Middle Pleistocene site (Spain). *Geobios*. **31**(5), 659-674 (1998).

[49] Blasco, R., Rosell, J., Peris, J. F., Cáceres, I. & Vergès, J. M. A new element of trampling: an experimental application on the Level XII faunal record of Bolomor Cave (Valencia, Spain). *J. Archaeol. Sci*. **35**(6),1605-1618 (2008).

[50] Santonja, M., Pérez-González, A., Panera, J., Rubio-Jara, S. & Méndez-Quintas, E. The coexistence of Acheulean and Ancient Middle Palaeolithic techno-complexes in the Middle Pleistocene of the Iberian Peninsula. *Quat. Int*. **411**, 367-377 (2016).

[51] Rios-Garaizar, J., Maidagan, D. G., Gomez-Olivencia, A., Iriarte, E., Arceredillo-Alonso, D., Iriarte-Chiapusso, M. J., Garcia-Ibaibarriaga, N., Garcia-Moreno, A., Gutierrez-Zugasti, I., Torres, T. & Aranburu, A. Short-term Neandertal occupations in the late Middle Pleistocene of Arlanpe (Lemoa, northern Iberian Peninsula). *C. R. Palevol*. **14**(3), 233-244 (2015).

[52] Gómez-Olivencia, A., Sala, N., Arceredillo, D., García, N., Martínez-Pillado, V., Rios-Garaizar, J., Garate, D., Solar, G. & Libano, I. The Punta Lucero Quarry site (Zierbena, Bizkaia): a window into the Middle Pleistocene in the Northern Iberian Peninsula. *Quat. Sci. Rev.* **121**, 52-74 (2015).

[53] Marks, A.E., Monigal, K., Chabai, V. P., Brugal, J. P., Goldberg, P., Hockett, B., Pemán, E., Elorza, M. & Mallol, C. Excavations at the Middle Pleistocene cave site of Galeria Pesada, Portuguese Estremadura: 1997–1999. *O Arqueólogo Português*. **20**, 7-39 (2002).

[54] Konidaris, G. E., Athanassiou, A., Tourloukis, V., Thompson, N., Giusti, D., Panagopoulou, E. & Harvati, K. The skeleton of a straight-tusked elephant (*Palaeoloxodon antiquus*) and other large mammals from the Middle Pleistocene butchering locality Marathousa 1 (Megalopolis Basin, Greece): preliminary results. *Quat. Int.* **497**, 65-84 (2018).

[55] Hoffecker, J. F., Baryshnikov, G. F. & Doronichev, V. B. Large mammal taphonomy of the Middle Pleistocene hominid occupation at Treugol’naya Cave (Northern Caucasus). *Quat. Sci. Rev.* **22**(5-7), 595-607 (2003).

[56] Baryshnikov, G. F. Pleistocene Canidae (Mammalia, Carnivora) from the Paleolithic Kudaro caves in the Caucasus. *Russ. J. Theriol*. **11**(2), 77-120 (2012).
